# High and selective cytotoxicity of ex vivo expanded allogeneic human natural killer cells from peripheral blood against bladder cancer: implications for natural killer cell instillation after transurethral resection of bladder tumor

**DOI:** 10.1186/s13046-024-02955-7

**Published:** 2024-01-20

**Authors:** Fangming Wang, Gang Zhang, Tianli Xu, Jianlin Ma, Jing Wang, Shuai Liu, Yuzhe Tang, Song Jin, Jianxing Li, Nianzeng Xing

**Affiliations:** 1 Department of Urology, Tsinghua University Affiliated Beijing Tsinghua Changgung Hospital, Tsinghua University Clinical Institute, Beijing, 102218 China; 2 BOE Regenerative Medicine Technology Co. Ltd, Beijing, 100015 China; 3 Department of Urology, National Clinical Research Center for Cancer/Cancer Hospital, National Cancer Center, Chinese Academy of Medical Sciences and Peking Union Medical College, Beijing, 100021 China; 4 State Key Laboratory of Molecular Oncology, National Cancer Center, National Clinical Research Center for Cancer/Cancer Hospital, Chinese Academy of Medical Sciences, Peking Union Medical College, Beijing, 100021 China

**Keywords:** Non-muscle-invasive bladder cancer, Natural killer cells, Transurethral resection of bladder tumor, Intravesical instillation, Chemokine

## Abstract

**Background:**

Non-muscle-invasive bladder cancer (NMIBC) is treated with transurethral resection of bladder tumor (TURBT) followed by intravesical instillation of chemotherapy or Bacillus Calmette–Guérin therapy. However, these treatments have a high recurrence rate and side effects, emphasizing the need for alternative instillations. Previously, we revealed that expanded allogeneic human natural killer (NK) cells from peripheral blood are a promising cellular therapy for prostate cancer. However, whether NK cells exhibit a similar killing effect in bladder cancer (BCa) remains unknown.

**Methods:**

Expansion, activation, and cryopreservation of allogeneic human NK cells obtained from peripheral blood were performed as we previously described. In vitro cytotoxicity was evaluated using the cell counting kit-8. The levels of perforin, granzyme B, interferon-γ, tumor necrosis factor-α, and chemokines (C-C-motif ligand [CCL]1, CCL2, CCL20, CCL3L1, and CCL4; C-X-C-motif ligand [CXCL]1, CXCL16, CXCL2, CXCL3, and CXCL8; and X-motif ligand 1 and 2) were determined using enzyme-linked immunosorbent assay. The expression of CD107a, major histocompatibility complex class I (MHC-I), MHC-I polypeptide-related sequences A and B (MICA/B), cytomegalovirus UL16-binding protein-2/5/6 (ULBP-2/5/6), B7-H6, CD56, CD69, CD25, killer cell Ig-like receptors (KIR)2DL1, KIRD3DL1, NKG2D, NKp30, NKp46, and CD16 of NK cells or BCa and normal urothelial cells were detected using flow cytometry. Cytotoxicity was evaluated using lactate dehydrogenase assay in patient-derived organoid models. BCa growth was monitored in vivo using calipers in male NOD-scid IL2rg−/− mice subcutaneously injected with 5637 and NK cells. Differential gene expressions were investigated using RNA sequence analysis. The chemotaxis of T cells was evaluated using transwell migration assays.

**Results:**

We revealed that the NK cells possess higher cytotoxicity against BCa lines with more production of cytokines than normal urothelial cells counterparts in vitro, demonstrated by upregulation of degranulation marker CD107a and increased interferon-γ secretion, by MICA/B/NKG2D and B7H6/NKp30-mediated activation. Furthermore, NK cells demonstrated antitumor effects against BCa in patient-derived organoids and BCa xenograft mouse models. NK cells secreted chemokines, including CCL1/2/20, to induce T-cell chemotaxis when encountering BCa cells.

**Conclusions:**

The expanded NK cells exhibit potent cytotoxicity against BCa cells, with few toxic side effects on normal urothelial cells. In addition, NK cells recruit T cells by secreting a panel of chemokines, which supports the translational application of NK cell intravesical instillation after TURBT from bench to bedside for NMIBC treatment.

**Supplementary Information:**

The online version contains supplementary material available at 10.1186/s13046-024-02955-7.

## Background

Bladder cancer (BCa) is the tenth most common cancer worldwide, with 573,278 cases and 212,536 related deaths in 2020 [1], imposing a substantial burden on healthcare systems [2].

According to pathologic tumor staging, BCa is classified into non-muscle-invasive bladder cancer (NMIBC) and muscle-invasive bladder cancer (MIBC) [3]. Among the most newly detected cases of BCa, approximately 75% correspond to patients with NMIBC [3–5] treated with transurethral resection of bladder tumor (TURBT) followed by an intravesical instillation of chemotherapy or Bacillus Calmette-Guérin (BCG) therapy, according to the risk stratification of progression. Regular cystoscopy, cytology, and some non-invasive approaches are used to follow up on patients with NMIBC [6]. The oncological control of NMIBC is far from satisfactory, with a 1-year and 5-year recurrence rate of 15–61% and 31–78%, respectively [7]. Moreover, 5–20% of NMIBC cases will eventually progress to MIBC, which remains incurable. Therefore, preventing NMIBC recurrence after TURBT is of utmost importance. Five recurrence mechanisms have been described for NMIBC: undetected tumors on cystoscopy, drop metastasis from upper tract urothelial carcinoma, incomplete resection during TURBT, tumor re-implantation after TURBT, and field-change cancerization [7, 8]. The first three mechanisms are influenced by clinicians before and during resection, and preventive agents can potentially influence the last three mechanisms. For example, intravesical chemotherapy instillation has been proposed to kill all floating cancer cells within the bladder and potentially any residual cancer cells in the resection bed [9]. Various agents employed for adjuvant therapies can be classified into two broad categories: chemotherapy and immunotherapy [10]. Chemotherapeutic agents such as epirubicin, mitomycin C, and gemcitabine kill BCa cells by interfering with the cell cycle [10]. The efficacy of chemotherapy is challenged by the difficulty in establishing a suitable and effective drug concentration because of the periodic discharge of the bladder. In addition, intravesical chemotherapy has significant side effects due to its nonspecific cytotoxicity to normal urothelial cells, limiting the doses that can be administered. Thus, tumor-specific treatments could reduce toxicity and enhance effectiveness by allowing higher doses [11]. Immunotherapeutic agents, such as BCG, induce a local immune response within the bladder but cannot directly kill BCa cells. Intravesical treatment with BCG is associated with more side effects than intravesical chemotherapy [12], and there are absolute contraindications to BCG therapy to avoid systemic absorption of the drug [3]. Additionally, BCG should be used with caution in immunocompromised patients [13]. Considering BCG’s severe side effects and limitations, another efficacious immunotherapy is needed to address these problems.

Natural killer (NK) cells are the predominant innate lymphocyte subset that mediates antitumor responses and possesses promising clinical applications for intravesical instillation for the following reasons: (1) NK cells have strong immunosurveillance against transformed cells and direct cytolytic activity against cancer cells without prior immune sensitization or major histocompatibility complex (MHC) restriction [14–16]; (2) The functionality of NK cells is modulated by an array of inhibitory and activating receptors that recognize their respective ligands on target cells [17], indicating that NK cells may exhibit selective cytotoxicity against cancerous and normal cells, contrary to conventional chemotherapy; (3) NK cells secrete cytokines and chemokines that subsequently shape the innate and adaptive immune response by promoting the recruitment of accessory immune cells, such as T cells, to the tumor site [18–20]; (4) We have mastered the NK cell expanding method and could obtain enough clinical therapeutic doses of NK cells [21]; and (5) The bladder is a hollow visceral organ that allows NK cells to interact directly with BCa and normal urothelial cells, indicating that NK cells can directly reach the tumor site with large-area contact and exert killing effects. In addition, NK cells and cytokines are restricted to the bladder and do not enter the bloodstream owing to local administration, thus causing minor side effects. Therefore, we propose that NK cell intravesical instillation could be exploited as a therapeutic strategy accompanied by TURBT in patients with NMIBC to prevent tumor recurrence with minor side effects, which has not yet been explored.

The current study aimed to validate expanded allogeneic peripheral blood-derived NK cells possess selective cytotoxicity against BCa and to reveal the exact profile of chemokines by which activated NK cells recruit T cells.

## Methods and materials

### Cell lines and culture

Human BCa cell lines (T24, UMUC-3, and 5637) and normal human urothelial cell line (SV-HUC-1) were obtained from the American Type Culture Collection. The human BCa cell line BIU-87 was obtained from the China Center for Type Culture Collection. BIU-87 and 5637 cells were cultured in RPMI-1640 medium (Servicebio) supplemented with 10% fetal bovine serum (FBS) (Biological Industries) and 1% penicillin/streptomycin (Solarbio, China). T24 and UMUC-3 were cultured in Dulbecco’s modified Eagle’s medium (HyClone) supplemented with 10% FBS. SV-HUC-1 cells were cultured in F-12 K medium (iCell-0007) supplemented with 10% FBS (Biological Industries) and 1% penicillin/streptomycin (Solarbio, China). All cell lines were cultured at 37 °C in a 5% CO_2_ standard incubator.

### NK and T-cell preparation

NK and T cells were obtained from the peripheral blood of healthy donors, as described previously [21, 22]. On day 0, after thawing, cryopreserved human peripheral blood mononuclear cells (PBMCs) were cultured statically for 24 h. Subsequently, the medium was supplemented with OpTmizer CTS T-cell expansion SFM (Invitrogen, MD) containing human IL-15, IL-2, OK432 (T&L Biology Technology Co. Ltd.). The PBMCs were inoculated into T75 flasks precoated with anti-human CD16 (BioLegend) (1 µg/mL) at a cell density of 2 × 10^6^ cells/mL. After 3–4 days of cultivation for activation, cells were transferred into the antibody-free culture flask in maintaining medium and cultured for 11 days. Fresh maintaining medium was added to the flask every 2–3 days according to the cell number, and the cell density was kept at 1 × 10^6^ cells/ml. All cell culture processes were performed at 37 °C in a humidified incubator with 5% CO_2_. On day 14, NK cells were obtained after CD3 negative and CD56 positive selection. T cells were obtained by CD56 negative and CD3 positive selection using magnetic beads (Miltenyi Biotec). The cell purity and count were evaluated using flow cytometry, and viability was assessed by acridine orange/propidium iodide (AO/PI) staining using an automatic fluorescence cell counter (Countstar).

### Cell counting kit-8 (CCK-8) assay

Target cells in the logarithmic growth phase were seeded in 96-well plates at 10,000 cells/well. On day two, NK cells were added at seven different effector-to-target ratios (E/T): 1.25:1, 2.5:1, 5:1, 10:1, 20:1, 40:1, and 80:1. Intravesical chemotherapeutic agents, including mitomycin and epirubicin, were added at two different concentrations: 0.5 and 1.0 mg/mL. Mixed incubations were performed for 0.5, 1, and 6 h. The killing rate was calculated as follows: killing rate = (OD_control_− OD_sample_/OD_control_−OD_medium_) × 100%.

The optical density (OD) was measured at 450 nm using an enzyme-linked immunosorbent assay (ELISA) plate reader (Multiskan FC; Thermo FC) as described previously [19].

### Degranulation assay

NK cell degranulation was evaluated based on CD107a expression in NK cells and the supernatant’s granzyme B and perforin-1 levels. NK cells were cocultured with or without BIU-87/T24/UMUC-3/5637/SV-HUC-1 cells (E/T = 10:1) for 1 h. The GolgiStop protein transport inhibitor (BD Biosciences) was added at the beginning of the coculture. After coculture, NK cells were collected, stained with anti-CD107a (LAMP-1) (BioLegend, H4A3) for 15 min, and analyzed using flow cytometry. Perforin-1 and granzyme B concentrations in the supernatants were measured using human ELISA kits for PRF1/PFP (CSB-E09313h) and granzyme B (CUSABIO, CSB-E08718h), respectively, following the manufacturer’s instructions.

### Cytokine and chemokine release assays

For the cytokine release assay, 1 × 10^6^ BCa or normal urothelial cells were plated in 6-well plates (Corning). After 12 h, the NK cells were added to the target cells at different E/T ratios. NK cells alone were used as the controls. The supernatant was collected after centrifugation at 0.5, 1, and 2 h. Interferon (IFN)-γ and tumor necrosis factor (TNF)-α concentrations in the supernatant were measured using human ELISA kits for IFN-γ (Invitrogen, KHC 4021) and TNF-α (CUSABIO, CSB-E04740h), respectively, according to the manufacturer’s instructions. The concentrations of interleukin (IL)-6 in serum collected from the inferior vena cava were measured using a human IL-6 ELISA Kit (Invitrogen) following the manufacturer’s protocol on day 28 after the subcutaneous tumor model mice were euthanized. For chemokine release assay in transwell experiment, concentrations of human chemokines in the supernatant including C-C-motif ligand 1 (CCL1), CCL2, CCL4, CCL20, C-X-C-motif ligand 1 (CXCL1), CXCL2, CXCL3, CXCL8, CXCL16, and C-motif ligand 1 (XCL1) were measured using human ELISA kits from CUSABIO for CCL1 (#CSB-EL004774HU), CCL2 (#CSB-Eq. 004783HU), CCL4 (#CSB-EL004797HU), CCL20 (#CSB-E04667h), CXCL1 (#CSB-E09150h), CXCL2 (#CSB-E07420h), CXCL3 (#CSB-EL006249HU), CXCL8 (#CSB-E04641h), CXCL16 (#CSB-E08871h), and XCL1 (#CSB-E08712h), while concentrations of human chemokine C-C-motif ligand 3 like protein 1 (CCL3L1) and XCL2 in the supernatant were measured using human ELISA kits for CCL3L1 (Cloud-Clone Corp, #SEA026Hu) and XCL2 (Cloud-Clone Corp, #SEC070Hu), respectively, following the manufacturer’s instructions.

### Flow cytometry

The BCa and normal urothelial cells (BIU-87/T24/UMUC-3/5637/SV-HUC-1) were stained with a combination of the following antibodies: anti-HLA-A, B, C (MHC class I [MHC-I]) (BioLegend, W6/32), anti-MHC-I polypeptide-related sequences A and B (MICA/B) (BioLegend, 6D4), anti-cytomegalovirus UL16-binding protein-2/5/6 (ULBP-2/5/6) (BioLegend, 165,903), and B7-H6 (Invitrogen, JAM1EW). The NK cells were stained with a combination of the following antibodies obtained from BioLegend: anti-CD56 (5.1H11), anti-CD69 (FN50), anti-CD25 (BC96), anti-CD158a (killer cell Ig-like receptors [KIR] 2DL1) (HP-DM1), anti-KIRD3DL1 (DX9), anti-NKG2D (1D11), anti-CD337 (NKp30) (P30-15), anti-CD335 (NKp46) (9E2), and anti-CD16 (3G8). T cells were stained with antibodies obtained from BioLegend: anti-CD181 (CXCR1)(8 F/CXCR1), anti-CD182 (CXCR2) (5E8/CXCR2), anti-CD186 (CXCR6)(K041E5), anti-CD191 (CCR1) (5F10B29), anti-CD192 (CCR2) (K036C2), anti-CD194 (CCR4) (L291H4), anti-CD195 (CCR5) (HEK/1/85a), anti-CD196 (CCR6) (G034E3), anti-CD198 (CCR8) (L263G8), and anti-XCR1 (S15046E). Cells were also stained with the corresponding isotype antibodies as controls. Data were acquired using a flow cytometer (LSR Fortessa; BD Biosciences). The results were analyzed using FlowJo software (version 10; Treestar, USA).

### Patient-derived organoids (PDO) culture and lactate dehydrogenase (LDH) assay

BCa and paracancerous tissue organoids were derived from the surgical samples of one patient with BCa at the Cancer Hospital, Chinese Academy of Medical Sciences. This study was approved by the Ethical Committees of the Tsinghua University Affiliated Beijing Tsinghua Changgung Hospital and Cancer Hospital, Chinese Academy of Medical Sciences (ethical approval number: NCC2021C-535). All patients who participated in this study provided signed informed consent. BCa and paracancerous tissue-derived organoids derived from a patient with BCa were established by K2 Oncology Co., Ltd. according to a previous report [23] and cultured in K2O-M-UB (K2 Oncology Co.) and K2O-M-UBN (K2 Oncology Co.) media, respectively. Organoid models were evaluated using a microscope for bright-field image analysis and hematoxylin and eosin (HE) staining for histomorphology analysis. The cytotoxicity of NK cells against PDOs was measured using a human LDH-Cytox Assay Kit (BioLegend, Cat#426,401) according to the manufacturer’s instructions. Briefly, 50 µL of target cell suspension was added to each well of a 96-well tissue culture plate (Corning 7007) (8,000 cells/well). Subsequently, 50 µL of cell culture medium was added to “high control” and “low control” wells, while 50 µL of cell culture medium containing NK cells was added at the E/T of 20:1. The mixed incubations were performed in a CO_2_ incubator for 2 h. Next, 10 µL of the lysis buffer was added to each “high control” well. The plate was incubated at 37 °C for 30 min in a CO_2_ incubator. Subsequently, 100 µL of the working solution was added to all wells and incubated at 25 °C for 30 min. The stop solution (50 µL) was added to all wells. The OD of the wells was measured at 490 nm using an ELISA plate reader (BMG Omega 7779). The cytotoxicity was calculated as follows: (OD_sample_−OD_low control_)/(OD_high control_−OD_low control_) × 100%.

### In vivo studies

Five-week-old male NOD-scid Il2rg−/− mice (NPGTM, VITALSTAR) were housed according to protocols approved by the Ethical Committee of the National Cancer Center (Ethical approval number: NCC2021C-535). We used a xenograft BCa mouse model to evaluate the antitumor effects of NK cells. The mice were subcutaneously injected with 5637 cells (1 × 10^7^ 5637 cells/mouse) mixed with either phosphate-buffered saline or NK cells (2.5 × 10^6^, 1 × 10^7^, or 1 × 10^8^). The mouse groups were labeled as (1) control, (2) NK, E/T = 0.25:1, (3) NK, E/T = 1:1, and (4) NK, E/T = 10:1 (*n* = 6 each). Tumor volumes were evaluated using calipers, as previously described [22]. All mice were euthanized on day 28, and the implanted tumors were harvested, photographed, weighed, and collected for histological examination. In addition, we established mouse satellite groups to monitor the survival time (*n* = 6 each).

### RNA sequencing and analysis

According to our previous method [20, 22], NK cells were obtained by CD56 positive and CD3 negative selection. Pure NK cells were cocultured with T24 cells for two hours (E/T = 10:1), and NK cells alone were used as controls. Viable NK cells were collected using fluorescence-activated cell sorting with negative PI staining. A minimum of 5 × 10^6^ cells was used for RNA analysis. RNA extraction and sequencing were performed as described previously [22]. Chemokine-encoding gene expression variations between cocultured and uncocultured NK cell populations were explored by differentially expressed gene analysis.

### Transwell chemotaxis assays

Isolated CD3^+^ T cells were suspended in an NK culture medium (3 × 10^5^ cells in 100 µL of medium) and seeded into the upper chambers of 24-well transwell plates (LABSELECT, 6.5-mm diameter and 5-µm pore size). Conditioned media collected from the culture of T24 cells alone, culture of NK cells alone, and coculture of NK cells with T24 cells (E/T = 10:1 for 2 h) were added to the lower chambers of the transwell plates, and the medium containing no cells was used as negative control. For blocking experiments, blocking antibodies against CCL1 (0.25 µg/mL; MAB272; R&D Systems), CCL2 (40 ng/mL; MAB679; R&D Systems), and CCL20 (10 µg/mL; MAB360; R&D Systems) were added into the conditioned media in the lower chambers. After 2 h of incubation at 37 °C, T cells were harvested from the lower chambers and stained with acridine orange/PI (Countstar). Cell counts were determined using a fluorescence cell analyzer (Countstar Rigel S2).

### Statistical analysis

All experiments were performed in triplicate, and the data are shown as the mean ± standard deviation (SD) or number (percentage). Comparisons of multiple groups were conducted using one-way analysis of variance followed by Tukey’s post hoc test; two independent groups were compared using Student’s t-test. For data that were not normally distributed, the Kruskal–Wallis and Mann–Whitney U tests were used to compare multiple and two groups, respectively. Categorical variables were analyzed using the χ2 test or Fisher’s exact test, as appropriate. The Kaplan–Meier method was used to plot survival curves; the log-rank test was applied to determine statistical significance for survival in xenogeneic experiments. Data analysis was conducted using SPSS 22.0 and GraphPad Prism 8 software. Two-sided *p* < 0.05 was considered statistically significant. The schematic figure was drawn with FigDraw (www.figdraw.com).

## Results

### Cytotoxicity of NK cells against human BCa cell lines and normal urothelial cells in vitro

**Fig. 1:**
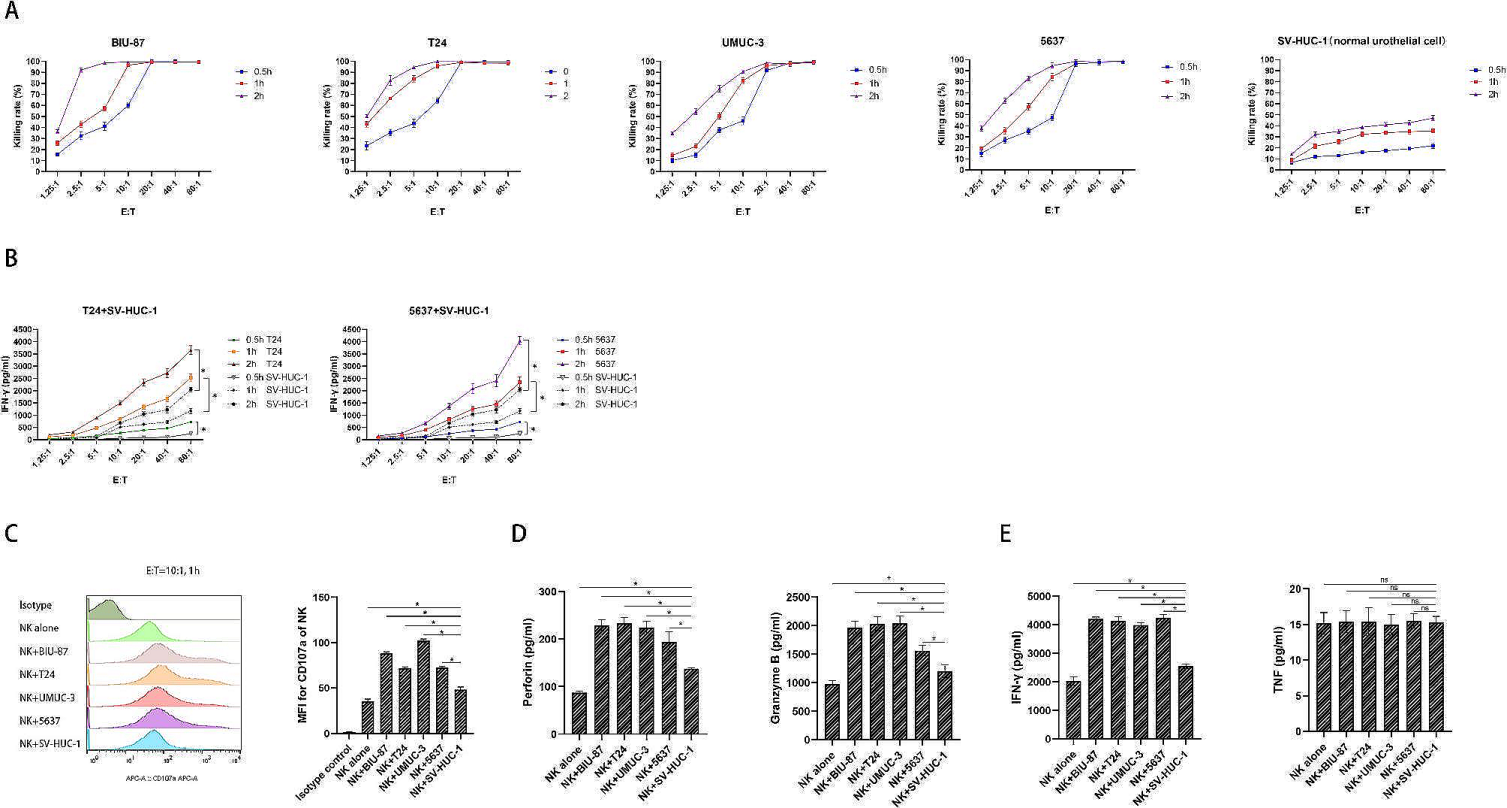
Cytotoxicity, degranulation activity, and cytokine secretion of the expanded NK cells. (**A**) Killing rates of the NK cells against multiple BCa cell lines (including BIU-87, T24, UMUC-3, and 5637) and normal epithelial cell line SV-HUC-1 at different E:T ratios after 0.5, 1, and 2 h coculture (*n* = 3); (**B**) Comparison of supernatant IFN-γ levels between NK cells cocultured with BCa cells (T24 or 5637) and their counterparts cocultured with normal epithelial cell line SV-HUC-1 at different E:T ratios after 0.5, 1, and 2 h coculture (*n* = 3); (**C**) Representative flow cytometry plots and summary data of CD107a expression on the NK cells after coculture with BCa cells, normal epithelial cells, and NK cells alone for 1 h (*n* = 3). E:T = 10:1; (**D**) Comparison of supernatant perforin and granzyme B levels between NK cells cocultured with BCa cells and their counterparts cocultured with normal epithelial cells at E:T of 10:1 after 1 h coculture (*n* = 3). NK cells alone were set as negative control. **E** Comparison of supernatant IFN-γ and TNF levels between NK cells cocultured with BCa cells and their counterparts cocultured with normal epithelial cells at E:T of 10:1 after 1 h coculture (*n* = 3). NK cells alone were set as negative control. Data expressed as means ± SD were plotted, and ANOVA followed by a Tukey’s post hoc test were used to compare three or more groups (**A–E**). *p < 0.05; ns, not significant. Abbreviations: BCa: bladder cancer; E:T: effector-to-target ratio; TNF: tumor necrosis factor

The CCK-8 results showed that NK cells exerted potent cytotoxicity against BCa cell lines, including BIU-87, T24, UMUC-3, and 5637, and the killing rate gradually increased with the elevation of E/T ratios (from 1.25:1 to 80:1) and elapsed coculture time (from 0.5 to 2 h) (Fig. 1A). The cytotoxic effects reached a peak at an E/T ratio of 20:1 and 1-hour coculture. The killing rate of NK cells against the normal urothelial cell line SV-HUC-1 was significantly lower than that against BCa cell lines at the corresponding E/T ratio and coculture time points (*p* < 0.05 for all comparisons). Our results demonstrated that the NK cells released more IFN-γ when cocultured with T24 or 5637 than with SV-HUC-1 at the same E/T ratio and coculture timepoint (*p* < 0.05 for all comparisons). In addition, the secretion of IFN-γ was positively correlated with the E/T ratio and coculture time (Fig. 1B). Furthermore, our data revealed that the expression of CD107a, a marker of NK cell degranulation, increased significantly in NK cells cocultured with BCa cell lines and normal urothelial cells compared with NK cells alone (E/T = 10:1, 1 h). CD107a expression in NK cells cocultured with SV-HUC-1 was significantly lower than that in NK cells cocultured with any BCa cells (Fig. 1C). The perforin, granzyme B, and IFN-γ levels in the supernatant from cocultures of NK and SV-HUC-1 were significantly higher than those in the supernatant from cocultures of NK cells and any BCa cells (Fig. 1D **and E**). We did not observe any significant difference in TNF-α levels among NK cells alone, cocultured with BCa cells, or normal urothelial cells (Fig. 1E). These data suggest that NK cells exhibit different functions of cytotoxicity and cytokine secretion when encountering BCa cells and normal urothelial cells, indicating the potential application of NK cell intravesical instillation.

### Cytotoxicity of mitomycin and epirubicin against human BCa cell lines and normal urothelial cells in vitro

**Fig. 2:**
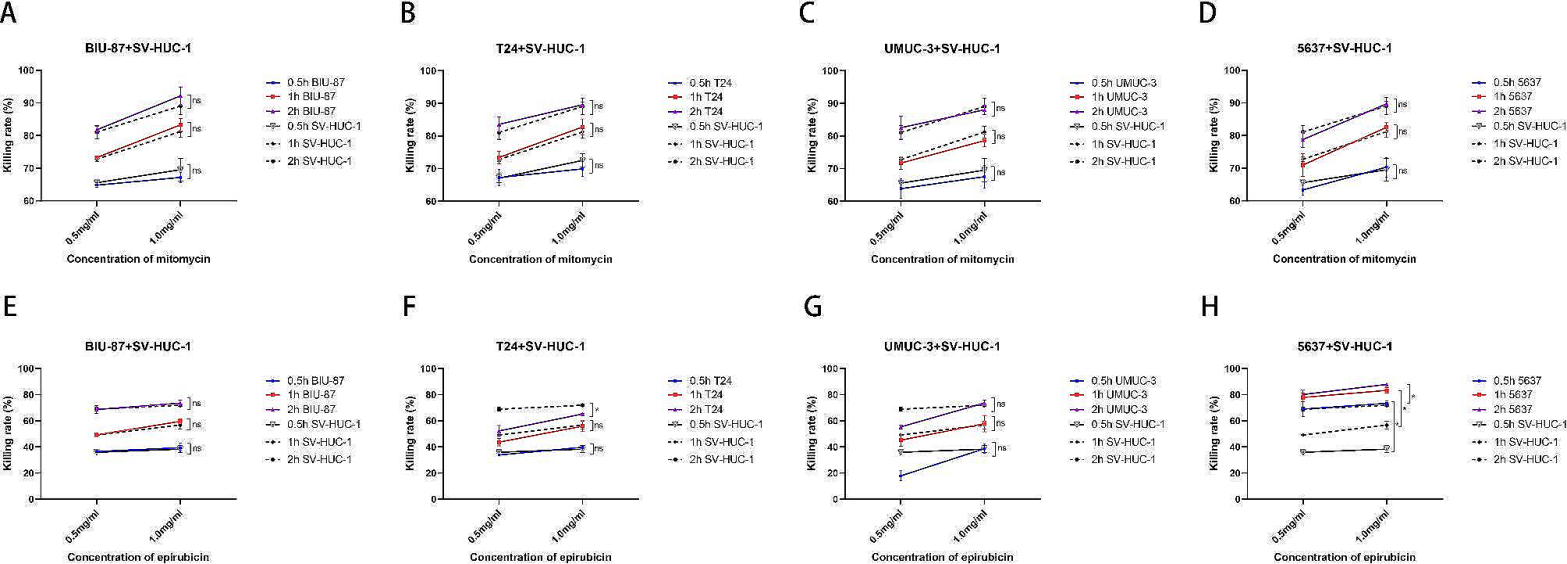
Comparison of cytotoxicity of chemotherapeutic agents against human BCa cell lines and normal urothelial cells *in vitro.* (**A–D**) Comparison of the killing rates of mitomycin at different concentration (0.5 and 1.0 mg/mL) against human BCa cell lines including BIU-87 (**A**), T24 (**B**), UMUC-3 (**C**), 5637 (**D**), and normal urothelial cell SV-HUC-1 measured using the CCK-8 assay at 0.5, 1, and 2 h (*n* = 3); **E–H** Comparison of killing rate of epirubicin at different concentration (0.5, 1.0 mg/mL) against human BCa cell lines including BIU-87 (**E**), T24 (**F**), UMUC-3 (**G**), 5637 (**H**), and normal urothelial cell SV-HUC-1 measured using the CCK-8 assay at 0.5, 1, and 2 h (*n* = 3). Data expressed as means ± SD were plotted, and Student’s t-test was used to compare two independent groups (A–H). *p < 0.05; ns, not significant

As shown in Fig. 2A–D, mitomycin showed no significant difference in the killing rate of all human BCa cell lines and normal urothelial cell SV-HUC-1 at 0.5 or 1.0 mg/mL and different time points (*p* > 0.05). For epirubicin treatment, the killing rate against 5637 was higher than that against SV-HUC-1 at 0.5, 1, and 2 h (*p* < 0.05), while the killing rate against T24 was lower than that against SV-HUC-1 at 2 h (*p* < 0.05). There was no significant difference between the killing rate against the other human BCa cell lines and SV-HUC-1 at 0.5 or 1.0 mg/mL at 0.5, 1, and 2 h (*p* > 0.05) (Fig. 2E–H). These data demonstrate that chemotherapeutic agents commonly used in clinical practice, such as mitomycin and epirubicin, exert no specific cytotoxicity against BCa and normal urothelial cells, explaining the side effects of adjuvant intravesical chemotherapy, such as urinary frequency, urgency, urodynia, and hemorrhage.

### Expression of activating and inhibitory receptors on NK cells and the corresponding ligands on BCa and urothelial cell lines

**Fig. 3:**
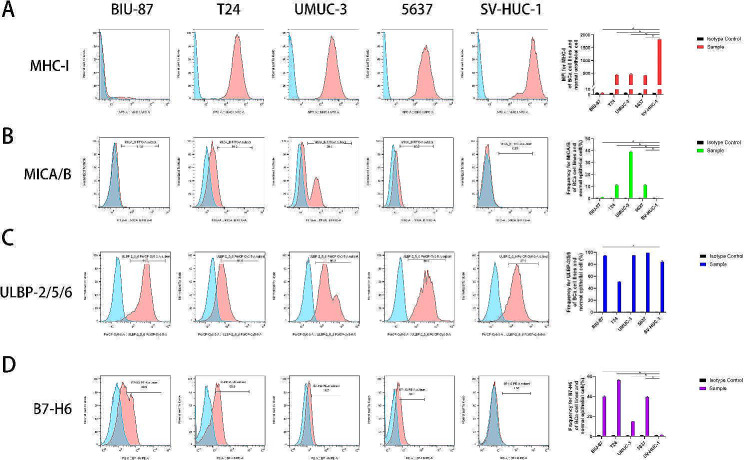
Expression of MHC-I, MICA/B, ULBP-2/5/6, and B7-H6 on surfaces of BCa and urothelial cells. (**A**) MHC-I expression on human BCa cell lines, including BIU-87, T24, UMUC-3, 5637, and normal urothelial cell line SV-HUC-1, were detected using flow cytometry. Representative images and summary data of the MFI for MHC-I are shown (*n* = 3); (**B**) MICA/B expression on human BCa cell lines and normal urothelial cell line SV-HUC-1 were detected using flow cytometry. Representative images and summary data of the percentages of MICA/B + cells are shown (*n* = 3); (**C**) ULBP-2/5/6 expressions on human BCa cell lines and normal urothelial cell line SV-HUC-1 were detected using flow cytometry. Representative images and summary data of the percentages of ULBP-2/5/6 + cells are shown (*n* = 3); (**D**) B7-H6 expression on human BCa cell lines and normal urothelial cell line SV-HUC-1 was detected using flow cytometry. Representative images and summary data of the percentages of B7-H6 + cells are shown (*n* = 3). All bars represent the means ± SD. Statistical significance was determined using an unpaired t-test (**A-D**). *p < 0.05. Abbreviations: MHC-I: major histocompatibility complex class I, MICA/B: MHC-I polypeptide-related sequences A and B, ULBP-2/5/6: cytomegalovirus UL16-binding protein-2/5/6; BCa: bladder cancer

**Fig. 4:**
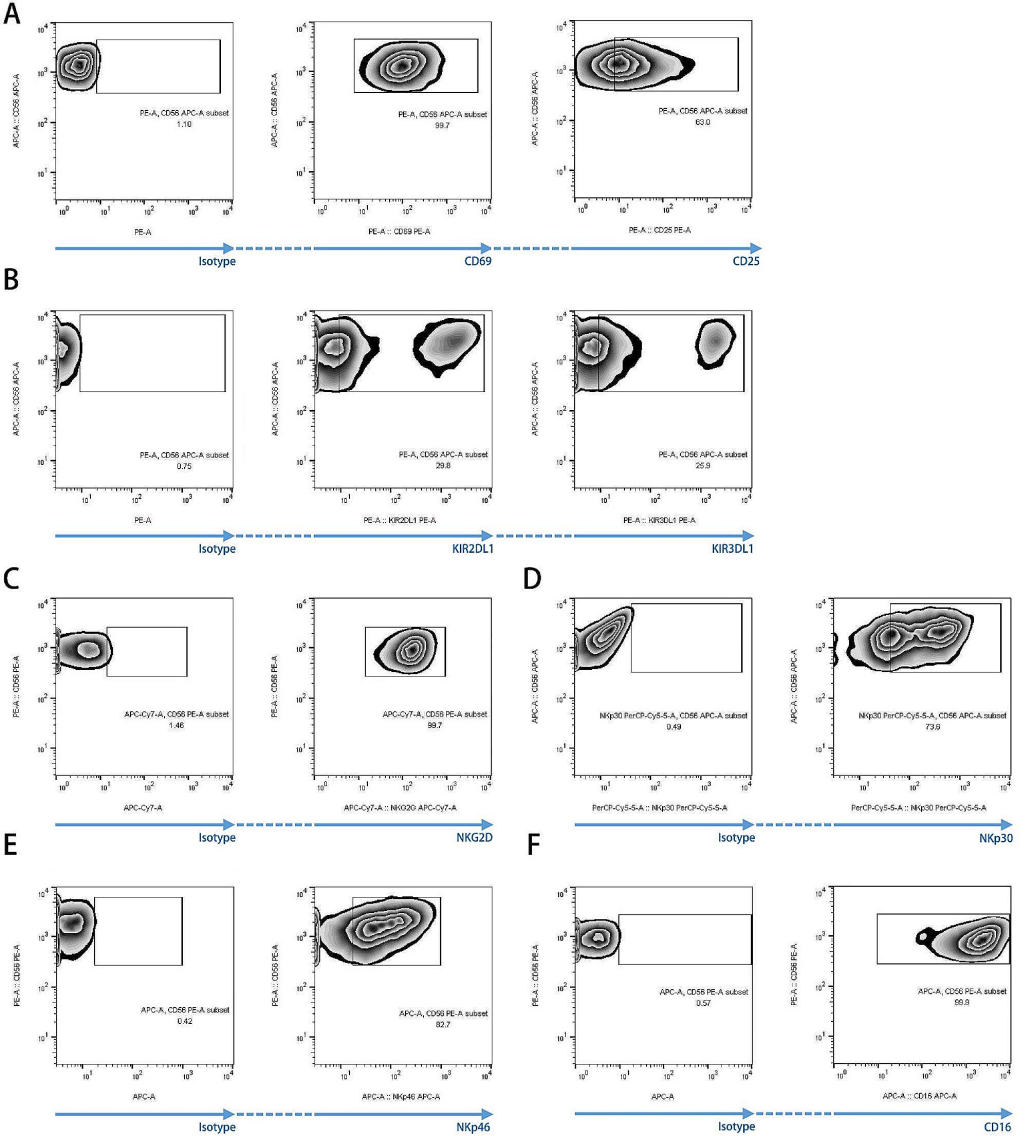
Expression of activating and inhibitory receptors on expanded peripheral blood-derived NK cells. The expressions of CD69 and CD25 (**A**), KIR2DL1 and KIR3DL1 (**B**), NKG2D (**C**), NKp30 (**D**), NKp46 (**E**), and CD16 (**F**) on NK cells were detected using flow cytometry (*n* = 3 for all molecules). Representative images are shown, respectively. Abbreviations: KIR: killer cell Ig-like receptor

To explore the mechanism of distinct cytotoxicity of NK cells against BCa and urothelial cells, we measured the expression of MHC-I, MICA/B, ULBP-2/5/6, and B7-H6 on BCa and urothelial cells. Our data showed that MHC-I expression was significantly lower in all BCa cell lines than in urothelial cells (Fig. 3A). Expression of MICA/B, a ligand of NKG2D, was significantly higher in BCa cells than in SV-HUC-1 cells (Fig. 3B). The expression of ULBP-2/5/6, another NKG2D ligand, was abundant in both BCa and urothelial cells (Fig. 3C). B7-H6 expression was negligible in urothelial cells but was abundant in all BCa cell lines (Fig. 3D). Subsequently, we measured the expression of activating and inhibitory receptors on NK cells. CD69 and CD25, NK cell activation markers, were highly expressed in the expanded NK cells (99.7% and 63%, respectively) (Fig. 4A). As the most important inhibitory and MHC-I receptors on NK cells, the following inhibitory KIRs were expressed in NK cells: KIR2DL1 (29.8%) and KIR3DL1 (25.9%) (Fig. 4B). As an activating receptor and ligand of MHC-I-like self-molecules, including MICA/B and ULBP-2/5/6, NKG2D was highly expressed in NK cells (99.7%) (Fig. 4C). NKp30, a natural cytotoxicity receptor and a receptor for B7-H6, was highly expressed in NK cells (73.6%) (Fig. 4D). NKp46, another natural cytotoxicity receptor, was also highly expressed in NK cells (82.7%) (Fig. 4E). In addition, CD16 was highly expressed in NK cells (99.9%) (Fig. 4F). In sum, our results demonstrate that allogeneic NK cells express an array of activating and inhibitory receptors, and BCa cells express much more the corresponding activating ligands than the normal urothelial cells, indicating the specific cytotoxicity of NK cells against BCa.

### NK cells repressed tumor development of PDO BCa models

**Fig. 5:**
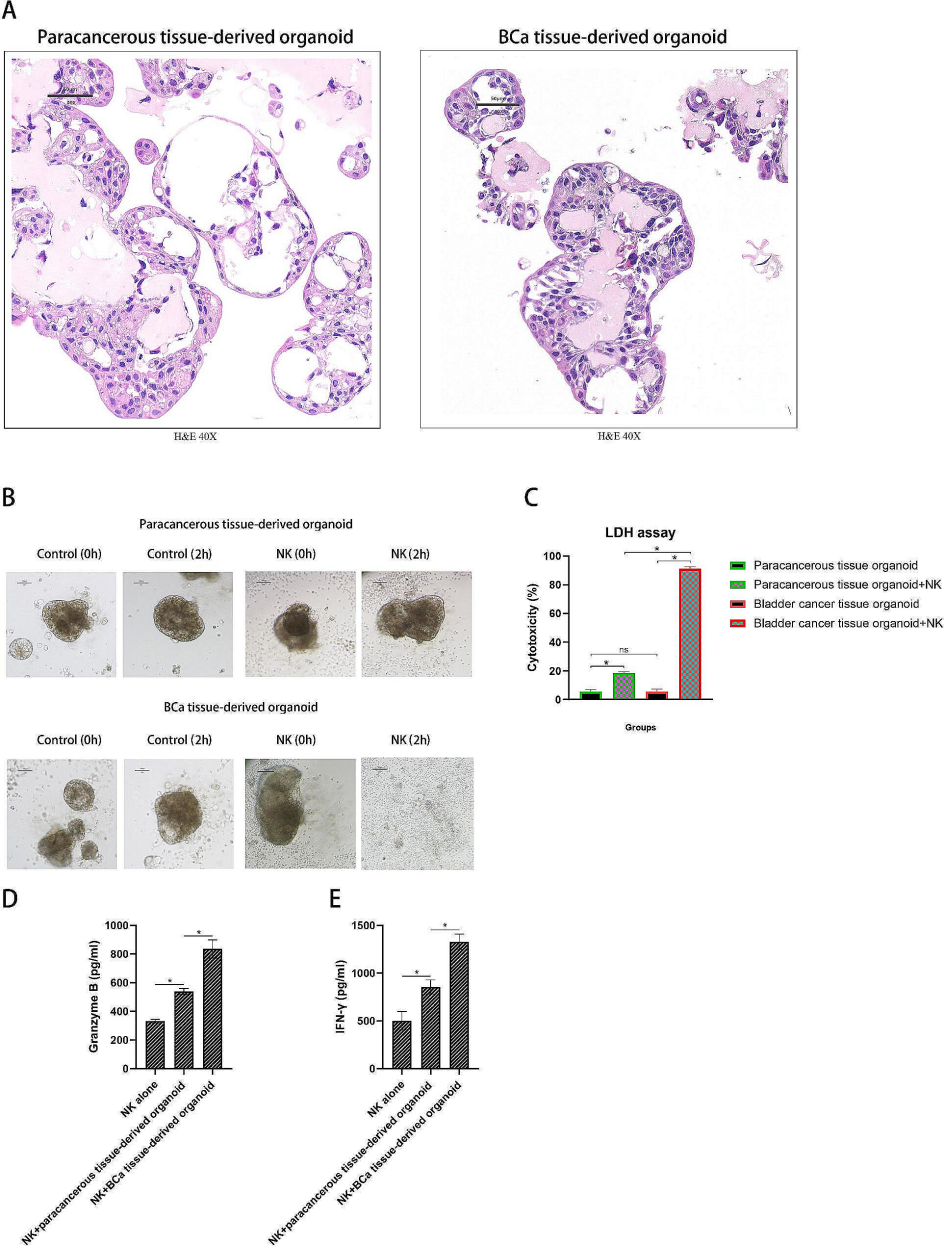
Development of human PDO BCa models and cytotoxicity of NK cells against the PDO. (**A**) Representative hematoxylin-and-eosin (H&E) images of paracancerous tissue-derived organoid and BCa tissue-derived organoid derived from BCa specimens (magnification ×40, scale bar 50 μm); (**B**) Representative bright-field image of coculture of NK cells with paracancerous tissue-derived organoid or BCa tissue-derived organoid, PDOs alone were set as controls (*n* = 3); (**C**) Cytotoxicity of NK cells against paracancerous tissue-derived organoid and BCa tissue-derived organoid at E/T ratio of 20:1 after 2 h coculture measured using LDH assay (*n* = 3); (**D**) Granzyme B levels of the supernatant after the NK cells were cocultured with paracancerous tissue-derived organoid and BCa tissue-derived organoid at E/T ratio of 20:1 after 2 h coculture measured using ELISA (*n* = 3); (**E**) IFN-γ levels of the supernatant after the NK cells were cocultured with paracancerous tissue-derived organoid and BCa tissue-derived organoid at E/T ratio of 20:1 after 2 h coculture measured using ELISA (*n* = 3). Data are shown as mean ± SD. Statistical significance was determined using an unpaired t-test (**C-E**). *p < 0.05; ns, not significant. Abbreviations: PDO: patient-derived organoid; BCa: bladder cancer; E/T: effector-to-target ratio; LDH: lactate dehydrogenase; ELISA: enzyme-linked immunosorbent assay

To evaluate the effects in models closer to the naïve tumor microenvironment (TME), we successfully established PDO in three-dimensional culture models using fresh tumor tissues from patients with BCa. HE staining (Fig. 5A) and RNA analysis (Supplementary material 1) confirmed that the PDO model retained pathological features of the BCa tumor tissue. The PDO models were treated with NK cells for two hours, generating potent antitumor effects against BCa tissue-derived organoids but low killing efficacy against paracancerous tissue-derived organoids, as observed in the bright-field image (Fig. 5B). The LDH assay results demonstrated that the cytotoxicity of NK cells against BCa tissue-derived organoids was much stronger than that against paracancerous tissue-derived organoids (*p* < 0.05) (Fig. 5C). Furthermore, we found that NK cells secreted much more granzyme B and IFN-γ when cocultured with BCa tissue-derived organoid than with paracancerous tissue-derived counterpart (*p* < 0.05 for both comparisons) (Fig. 5D **and E**). Altogether, these results indicate that NK cells displayed specific cytotoxicity against BCa tissues directly derived from patients, further supporting the clinical applicability of NK cell intravesical instillation.

### NK cells control the growth of locally implanted BCa

**Fig. 6:**
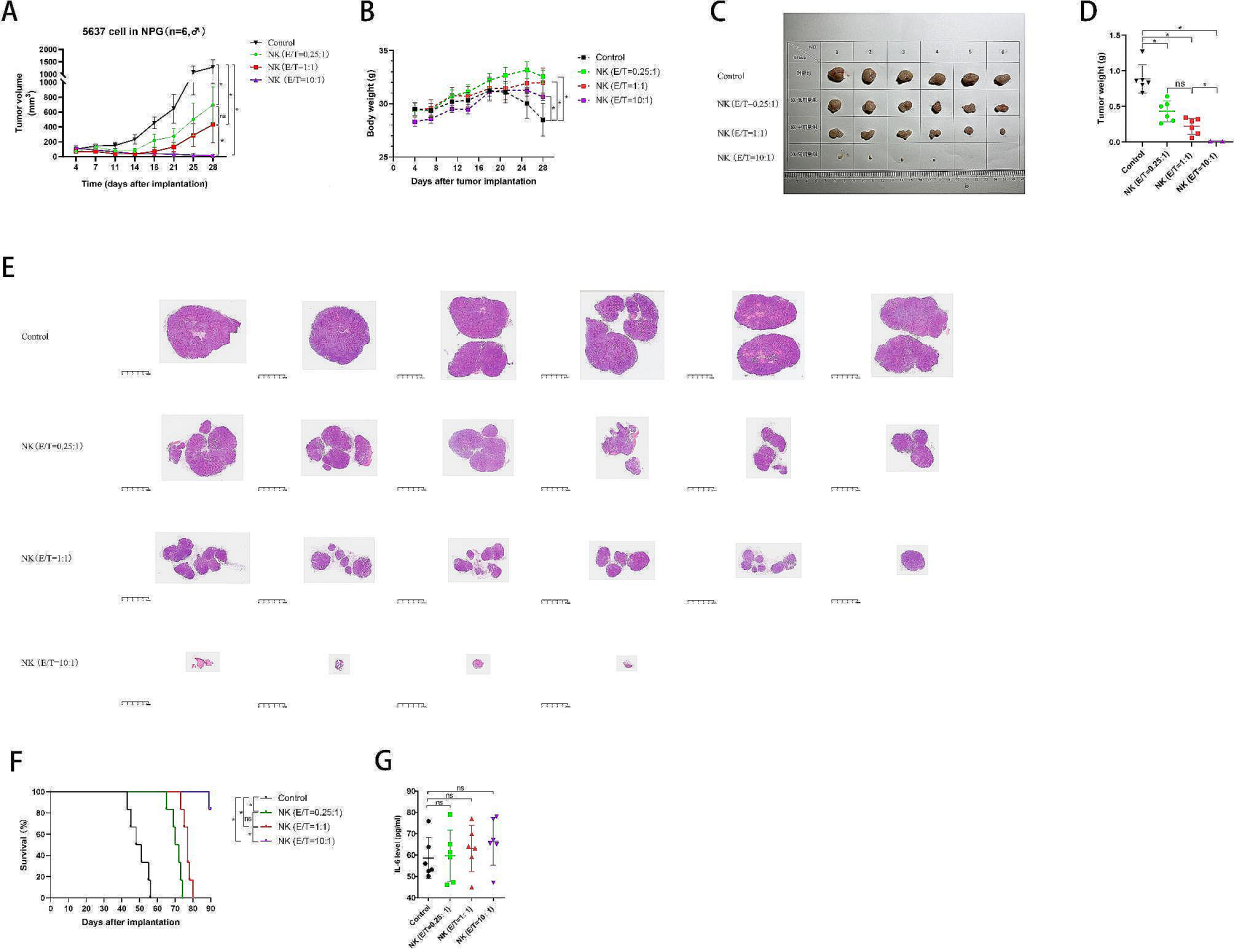
Antitumor effect of NK cells against BCa in a subcutaneous tumor model in vivo. (**A**) Tumor volumes at various times (horizontal axis) after tumor implantation in the control and treatment groups, including NK (E/T = 0.25:1), NK (E/T = 1:1), and NK (E/T = 10:1) groups. Tumor volumes were calculated according to the formula L×W^2^/2, where L and W represent the longest and shortest diameters measured using a caliper, respectively (*n* = 6 per group); (**B**) Body weights in the control and treatment groups over the whole treatment course (*n* = 6 per group); (**C**) Images of tumors in mice 28 days after tumor implantation (*n* = 6 in each group); (**D**) Tumor weights corresponding to each group when harvested on day 28 (*n* = 6 in each group); (**E**) HE examination of tumor specimen in the control and treatment groups on day 28 (*n* = 6 or 4 for each group); (**F**) Cumulative Kaplan–Meier survival curves for mice (*n* = 6 per group) after tumor implantation; (**G**) Serum IL-6 levels in the control and NK cell treatment groups on day 28 (*n* = 3, each). Data expressed as mean ± SD were plotted, and ANOVA followed by a Tukey’s post hoc test was used for multiple group comparisons (**A**, **B**, **C**, **D** and **G**). The Kaplan–Meier method was used to estimate survival functions. The log-rank test was used for group comparisons (**F**). *p < 0.05; ns, not significant. Abbreviations: BCa: bladder cancer; E/T: effector-to-target ratio; HE: hematoxylin-and-eosin; IL-6: interleukin-6

Subcutaneous co-injection of NK and BCa cells could partly recapitulate the actual therapeutic setting of NK cell instillation for treating BCa recurrence after TURBT. When NK cells were co-injected subcutaneously with 5637 cells, NK cells significantly delayed the tumor growth of BCa compared with the control group; this therapeutic effect was evidently and positively correlated with the E/T ratios (Fig. 6A). The mouse body weight showed no significant difference among the four groups before day 20; however, it was higher in the treatment groups than in the control group after day 20 (Fig. 6B). After harvesting the tumors from mice on day 28, we photographed and weighed the tumors; the tumor masses in the NK cell groups were lower than those in the control group (Fig. 6C **and D**). At an E/T ratio of 10:1, tumor growth was completely blocked, and no neoplastic mass developed in two mice (Fig. 6C). HE staining of the tumor specimens was consistent with the tumor weight results. It was noteworthy that the tumor was separated into more small lobes as the E/T ratio increases from 0, 0.25:1 to 1:1. However, when the E/T ratio reached to 10:1, the number of tumor lobes decreased evidently and the tumor was reduced to one small mass (Fig. 6E). Moreover, mice treated with NK cells survived longer than those treated with phosphate-buffered saline (Fig. 6F). Furthermore, the serum IL-6 levels of mice in the NK cell treatment groups were slightly higher than those in the control group but not statistically significantly different (*p* = 0.595) (Fig. 6G). Overall, these findings indicate that NK cells exert therapeutic effects against BCa tumors in mice used to simulate the actual human therapeutic setting, providing solid support for the clinical application of NK cell instillation.

### NK cells drive T-cell recruitment by secreting CCL1/2/20 in vitro

**Fig. 7:**
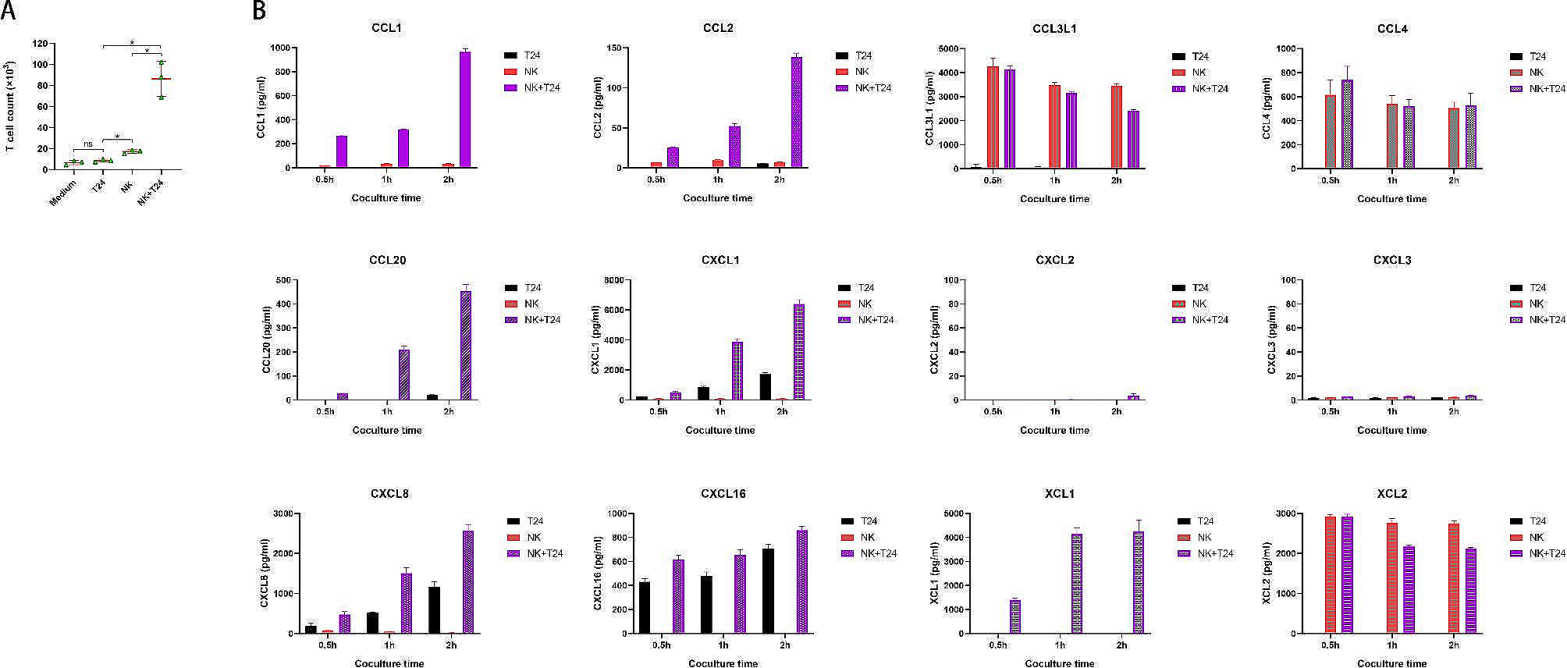
The number of T cells migrating across the transwell and the protein expression levels of chemokines mediating T-cell recruitment. (**A**) The activated CD3^+^ T cells were seeded into transwells with simple media and conditioned media from T24 cell cultures, NK cell cultures, and NK and T24 cell cocultures. Summary data of the cumulative numbers of CD3^+^ T cells that migrated across the transwell in each condition are shown (*n* = 3 per group). ns, not significant; *p < 0.05 ; (**B**) Protein expression levels of chemokines in the supernatant from T24 culture, NK culture, and NK + T24 coculture measured using ELISA (*n* = 3 per group). Data expressed as mean ± SD were plotted. Abbreviations: CCL: C-C-motif ligand; CXCL:C-X-C-motif ligand; XCL1:X-C-motif ligand

**Fig. 8:**
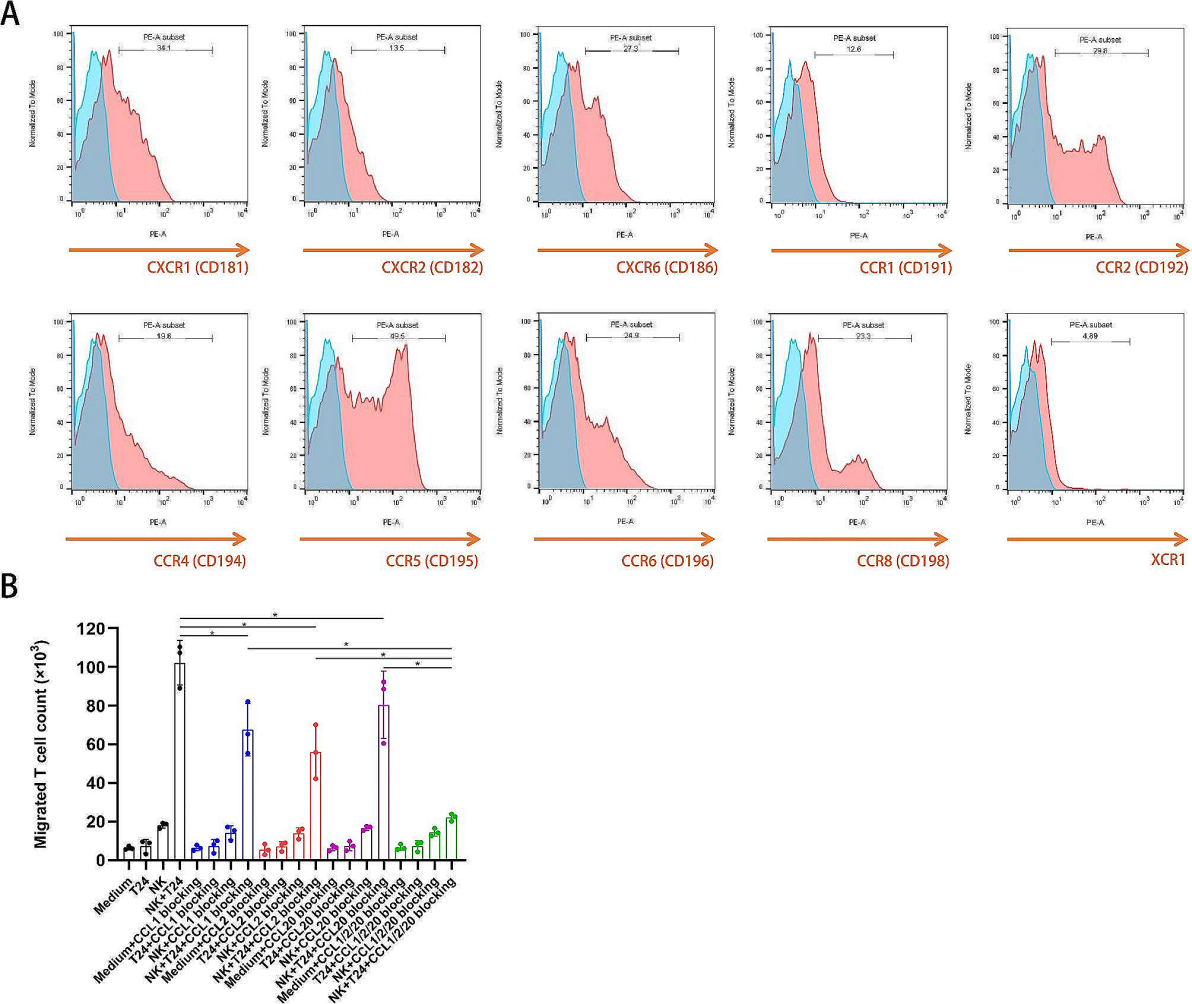
Receptors of the chemokines expressed on CD3^+^ T cells and T cell count migrating across the transwell after blocking CCL1/2/20 in the supernatant of coculture medium. (**A**) The expressions of the chemokine receptors on CD3^+^CD56^−^ T cells were detected using flow cytometry (*n* = 3 for all molecules). The representative images are shown. (**B**) Summary data of the cumulative numbers of T cells determined using a fluorescence cell analyzer after being harvested from the lower chambers, which were added into blocking antibodies against CCL1 (0.25 µg/mL), CCL2 (40 ng/mL), or CCL20 (10 µg/mL) (*n* = 3 for each). Data are shown as mean ± SD. Statistical significance was determined using an unpaired t-test. *p < 0.05. Abbreviations: CXCR: C-X-C chemokine receptor; CCR:C-C chemokine receptor; XCR:X-C chemokine receptor

To determine the T-cell recruitment driven by the coculture of NK and BCa cells, we performed transwell experiments using our previously described method [20]. Migration assays indicated that T-cell migration through the transwell was low in the control and T24-conditioned media. Evident migration was observed in the NK cell-conditioned medium (*p* < 0.05), and migration was significantly enhanced by conditioned media from the NK and T24 cell cocultures (Fig. 7A). To identify the chemokines produced by NK cells that promote T-cell migration, we performed RNA sequencing of NK cells. There were 1,140 upregulated and 196 downregulated genes when comparing NK cells cocultured with T24 cells with their uncocultured counterparts (Supplementary materials 2 and 3). Notably, genes encoding the chemokines CCL1, CCL2, CCL20, CCL3L1, CCL4, CXCL1, CXCL16, CXCL2, CXCL3, CXCL8, XCL1, and XCL2 were significantly upregulated in NK cells cocultured with BCa cells (labeled in blue in Supplementary material 2). The protein expression levels of these genes were measured in the supernatant using ELISA to identify the chemokines mediating T-cell recruitment. The expression data for CCL1, CCL2, CCL20, and XCL1 were consistent with the transwell results, whereas the expression data for CCL3L1, CCL4, CXCL1, CXCL2, CXCL3, CXCL8, CXCL16, and XCL2 were inconsistent with the migration results (Fig. 7B). We also examined the corresponding chemokine receptors on CD3^+^T cells. The data demonstrated that the expression frequencies of CXCR1 (receptor of CXCL8), CXCR2 (receptor of CXCL1, 2, 3, and 8), CXCR6 (receptor of CXCL16), CCR2 (receptor of CCL2), CCR4 (receptor of CCL2), CCR5 (receptor of CCL4 and CCL3L1), CCR6 (receptor of CCL20), and CCR8 (receptor of CCL1, 4) were 34.1%, 13.5%, 27.3%, 29.8%, 19.6%, 49.5%, 24.9%, and 23.3%, respectively (Fig. 8A). The expression levels of receptors of preliminarily screened chemokines (CCL1, CCL2, CCL20, and XCL1) were as follows: CCR8 (23.3%), CCR2 (29.8%), CCR4 (19.6%), CCR6 (24.9%), and XCR1(4.89%). Because XCR1 expression in T cells was negligible, we ruled out the XCL1/XCR axis and focused on CCL1, CCL2, and CCL20. To validate the screened chemokines responsible for T-cell chemotaxis, we performed additional transwell assays with neutralizing antibodies against CCL1, CCL2, and CCL20. As shown in Fig. 8B, CCL1, CCL2, and CCL20, neutralization significantly reduced the number of migrated T cells in the cell-conditioned media of the NK + T24 coculture group. Blocking antibodies against CCL1, CCL2, and CCL20 decreased the number of T cells in the coculture group to a level close to that in the NK cells alone group, indicating that combinations of these chemokines contribute overwhelmingly to mediate T-cell recruitment. Taken together, we screened all possible chemokines secreted by NK cells and corresponding receptors on T cells, and finally identified that the cytokine network involving CCL1, CCL2, and CCL20 are responsible for promoting T-cell recruitment.

## Discussion

We demonstrated that expanded NK cells derived from human peripheral blood possessed potent cytotoxicity against BCa cells in vitro and PDO and xenograft mouse models of BCa in vivo. The cytotoxicity of NK cells against BCa cells was much higher than that against normal urothelial cells, different from chemotherapy that caused similar cytotoxicity against the two types of cells. Herein, we revealed the molecular mechanisms by which allogeneic NK cells exhibit high and selective cytotoxicity against BCa cells. We identified the chemokines by which highly activated NK cells induce T-cell chemotaxis. This study is the first to comprehensively explore the antitumor efficacy and mechanisms of NK cell therapy against BCa and identify the chemokines responsible for T-cell recruitment. Our study supports the clinical application of NK cell instillation after TURBT for NMIBC treatment.

BCa is a major urological disease, with approximately 550,000 new cases diagnosed yearly worldwide [1, 24]. Urothelial carcinoma accounts for over 90% of all bladder cancers [25]. In total, 75% of patients with BCa present a disease confined to the mucosa or submucosa, known as NMIBC [3–5]. TURBT, a minimally invasive surgery, is a standard procedure in NMIBC treatment in which all visible tumors within the bladder are resected without breaching and opening up the bladder wall. Although TURBT is a potentially curative surgery, a high NMIBC recurrence rate has been observed [7, 8, 26]. Treatment improvements have based on addressing the causes of recurrence; for instance, computed tomography urogram examination for upper tract urothelial carcinoma detection, advances in endoscopic imaging techniques for BCa detection and surgical techniques, and intravesical BCG therapy or chemotherapy have been used [7]. However, despite recent advances in NMIBC treatment, oncological outcomes remain unsatisfactory [27, 28]. Intravesical chemotherapy or BCG therapy, involving direct transurethral administration of the therapeutic agent(s) into the bladder via a catheter, is usually used in an attempt to extend the recurrence-free interval after surgery in clinical settings but has limited efficacy and severe side effects such as bladder irritation symptoms and even systemic infection [10–12]. We have previously demonstrated that high doses of NK cells exhibit antitumor effects against refractory prostate cancer [21]. We speculated that high doses of NK cells could have a similar therapeutic effect on MIBC but with severe side effects. NK cell engineered with chimeric antigen receptor (CAR) targeting specific antigens on BCa cells could be a promising approach for treating MIBC. However, NK cells may be candidates for intravesical instillation for treating NMIBC after TURBT considering factors such as bladder anatomy, characteristics of NK cells (especially their activation mechanism and recruitment effects), and availability of a clinical dosage of NK cells. NK cells can kill target cells via degranulation, expression of Fas ligand, and TNF-related apoptosis-inducing ligand (TRAIL). In addition to their cytotoxic capacity, NK cells can secrete multiple cytokines, including IFNγ and TNF, to influence the activity of other immune cells [29].

Based on the above inference, we first validated that the expanded NK cells exerted potent cytotoxicity against diverse human BCa cell lines, including BIU-87, T24, UMUC-3, and 5637, particularly at higher E/T ratios (≥ 10:1) after one hour. We made an important discovery: the cytotoxicity of NK cells against normal urothelial cells was significantly lower than that against BCa cell lines at the same E/T ratio and coculture timepoint, supported by a higher upregulation of CD107a expression and release of effector molecules, including perforin and granzyme B. Moreover, the IFN-γ production of NK cells cocultured with BCa or normal cells was consistent with the cytotoxicity assay results. NK cells have been recognized as the primary producers of IFN-γ in many physiological and pathological conditions [19]. IFN-γ promotes T-helper 1 cell polarization, induces MHC class II molecules on antigen-presenting cells and activates macrophages [29]. Our results indicate that IFN-γ produced by NK cells after killing BCa cells would help to reshape the immune profile in the TME and make TME hotter. TNF-α is a strong pro-inflammatory cytokine that plays an important role in the immune system during inflammation, cell proliferation, differentiation and apoptosis [30]. Our previous study tested the TNF levels after the NK cells were cocultured with prostate cancer cells at E/T ratio of 10:1 for 12 h, and we observed evident TNF-α secretion [21]. The insignificant difference in TNF-α level among groups of NK alone, NK cocultured with BCa or normal cells may be due to the short coculture time in our study. The low secretion of TNF-α after 1 h in our study indicates that NK cell therapy could not cause severe inflammatory reaction, such as cytokine release syndrome (CRS), a side effect of adoptive cell therapy. The differential cytotoxicity of NK cells against BCa cell lines and normal urothelial cells indicates that NK cell intravesical instillation could efficiently eliminate the residual BCa cells and any emerging aberrant cells after TURBT while causing relatively little damage to normal urothelial cells. The in vitro results strongly suggest that an immunotherapeutic strategy using allogeneic activated NK cells is effective with negligible side effects. In contrast, we found that the chemotherapeutic agents mitomycin and epirubicin exerted similar cytotoxicity against BCa cells and normal urothelial cell lines, completely different from the differential cytotoxicity of NK cells against target cells. Mitomycin is a commonly used intravesical cytotoxic agent, and its mechanism of action is primarily alkylation and the subsequent cross-linking of DNA strands, resulting in cell death [31, 32]. Farr et al. reported that mitomycin exhibited equivalent cytotoxic potency in normal and malignant bladder urothelial cultures [33]. Similarly, Huo et al. reported that the intravesical instillation of epirubicin lacks tumor selectivity for treating BCa and often causes severe damage to the normal bladder urothelium, leading to intolerable side effects [34]. These reports are consistent with our results, indicating a lack of selectivity of chemotherapeutic drugs. Taken together, these results validated that.

NK cells are cytotoxic to malignant but not non-malignant urothelial cells. Although pre-clinical, our results strongly suggest that an immunotherapeutic strategy using allogeneic activated NK cells from healthy donors is effective and should be exploited as a complementary therapeutic strategy in NMIBC patients to prevent tumor recurrence and progression.

**Fig. 9:**
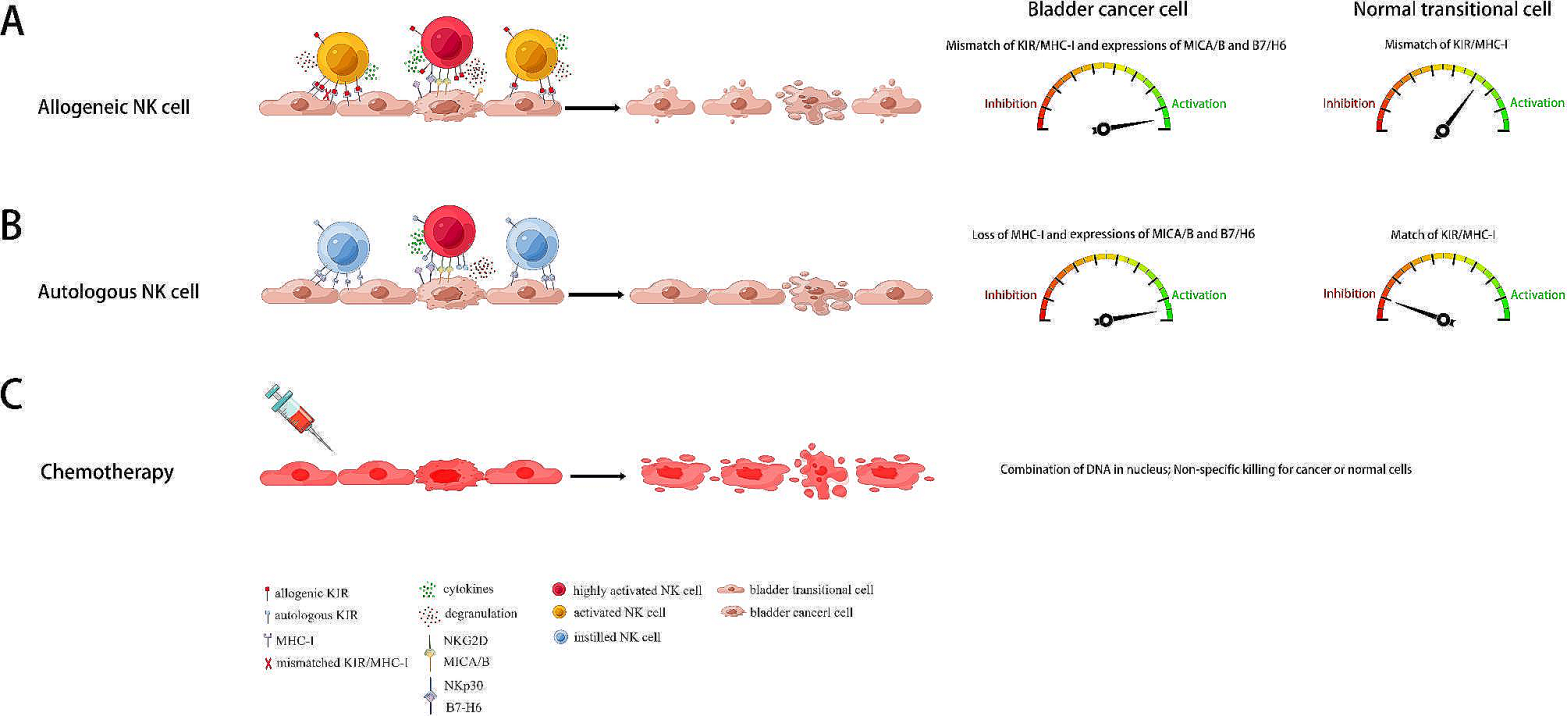
Summary diagram of cytotoxicity mechanisms of intravesical instillation of NK cells and chemotherapy. (**A**) The mechanisms of different cytotoxicity of allogeneic NK cells against BCa and normal transitional cells. When encountering BCa cells, allogeneic NK cells were strongly activated through a mismatch of inhibitory KIR/MHC-I, activating MICA/B/NKG2D and B7H6/NKp30 signaling pathways. On the contrary, when encountering normal transitional cells, allogeneic NK cells were activated only through a mismatch of inhibitory KIR/MHC-I. (**B**) The mechanisms of different cytotoxicity of autologous NK cells against BCa and normal transitional cells. When encountering BCa cells, autologous NK cells were activated through relief of KIR/MHC-I inhibition due to loss of MHC-I on BCa cells, activating MICA/B/NKG2D and B7H6/NKp30 signaling pathways. On the contrary, when encountering normal transitional cells, autologous NK cells were inhibited through a match of the inhibitory KIR/MHC-I signaling pathway. (**C**) Chemotherapy exerts nonspecific cytotoxicity against BCa and normal cells by combining DNA in the nucleus. Abbreviations: BCa: bladder cancer; KIR: killer cell Ig-like receptor; MHC-I: major histocompatibility complex class I; MICA/B: MHC-I polypeptide-related sequences A and B; NKG2D: NK group 2 member D

We explored the high and selective cytotoxicity mechanism of NK cells against BCa cells. As the main effector cell type in innate immunity, NK cells are capable of killing tumor cells at a very early stage and rely on the “missing self” and “induced self” modes to identify target cells by maintaining a precise balance between activating co-stimulatory and inhibitory signals [35]. The “missing self” mode alone is not enough to protect tumor cells from being killed by NK cells. Studies have reported that the susceptibility of BCa cells does not entirely depend on “missing self”-recognition, and NK cell activation induced by activating ligands is a powerful mechanism to overcome MHC class I inhibitory signals [36, 37]. Our data revealed that KIRs, the most important inhibitory and MHC-I receptors on NK cells, were widely expressed in NK cells. All BCa cell lines except BIU-87 expressed MHC-I on their surfaces. However, the levels of MHC-I were significantly lower than those of urothelial cells, indicating that the “induced self” mode contributes to NK cell activation. In our study, the MICA/B (the ligand of NKG2D) and B7-H6 (the ligand of NKp30) were highly expressed on most BCa cell lines, with an extremely low expression on normal urothelial cells. The corresponding receptors NKG2D and NKp30 were highly expressed on NK cells, allowing NK cells to efficiently recognize the malignant cells by the “induced self” mode. The expression of ULBP-2/5/6, another ligand of NKG2D, was abundant on both BCa and urothelial cells and may also activate NK cells by “induced self” mode. Moreover, NKp46, a highly expressed activating receptor on NK cells, may bind to unidentified ligands specifically expressed in BCa cells. In summary (Fig. 9), allogeneic NK cell intravesical therapy exerts high cytotoxicity against BCa cells due to the mismatch of KIR/MHC-I and highly expressed activating ligands, including MICA/B and B7-H6, on BCa cells but low cytotoxicity against normal urothelial cells due to the mismatch of KIR/MHC-I only. In contrast, autologous NK cells exert high cytotoxicity against BCa cells owing to the loss or downregulation of MHC-I and highly expressed activating ligands on BCa cells, but no cytotoxicity against normal urothelial cells owing to inhibitory signal transduction by the matched KIR/MHC-I. Moreover, chemotherapy caused similar cytotoxicity to BCa and normal cells.

Although the BCa patient-derived cell lines used in our study originated from tumors of different stages and histologic variants and had most of the genetic aberrations of the original tumor [38, 39], we further developed BCa PDO models to validate the high and selective cytotoxicity of NK cells against BCa cells. PDOs can accurately recapitulate tumor tissue architecture and have thus been successfully used as functional models for predicting drug response in different cancers [40]. In this study, we simultaneously and successfully established BCa and paracancerous tissue-derived organoids. We found that NK cell treatment produced potent antitumor effects against BCa tissue-derived organoids but not against paracancerous tissue-derived organoids. This is consistent with our in vitro cell line data and provides further evidence for the NKs’ clinical use. Furthermore, we used a xenograft BCa mouse model to evaluate the antitumor effects of NK cells in vivo. We subcutaneously administered BCa cells with or without freshly expanded NK cells at different ratios. NK cell therapy efficiently impeded PCa growth, further confirmed by the weight of the harvested tumor specimens. The HE staining could accurately describe the morphological architecture of the harvested tumors and also evaluate the efficacy of NK cell therapy. The HE staining results indicates that the BCa cells were separated into small “islands” surrounded by NK cells and presented multi-point growth after NK cell treatment. When the E/T ratio was increased, the multi-point growth was inhibited and only 1–2 “islands” were left. In addition, the mice treated with NK cell therapy survived longer than their counterparts without NK cell therapy. Compared to the control group, NK cell therapy did not significantly increase serum IL-6 level, a representative cytokine that causes severe CRS. Overall, our study indicated that the administration of NK cells displayed significant therapeutic efficacy against BCa in vivo without evident side effects, which aligns with the in vitro results. Our results in vivo are consistent with those of a previous study [37], which reported that the adoptive transfer of healthy activated NK cells displayed antitumor activity in mice bearing cancer stem-like cell-induced orthotopic BCa. Although that study did not explore the selective cytotoxicity of NK cells against BCa, our conclusions were similar. In this study, we used immunocompromised mice lacking T cells, which play a central role in tumor surveillance. NK cells can recruit T cells and other immune cells and interact with them through positive feedback. Therefore, the significant results we obtained in immunocompromised mice are likely to underestimate the therapeutic efficacy of NK cells in immunocompetent hosts.

This study evidenced that NK cells can recruit T cells in vitro while killing BCa cells, which was supported by our previous study [20] and Cichocki et al. [41]. To further explore the exact chemokines by which NK cells recruit T cells, we performed RNA sequencing and differentially expressed gene analysis, which revealed that the expression of CCL1, CCL2, CCL20, CCL3L1, CCL4, CXCL1, CXCL16, CXCL2, CXCL3, CXCL8, XCL1, and XCL2 was markedly upregulated in activated cocultured NK cells compared with their quiescent uncocultured counterparts. Furthermore, we tested the levels of these chemokines in supernatants using ELISA. After comprehensive analysis of all chemokine secretions and transwell assays, we confirmed that CCL1, CCL2, CCL20, and XCL1 were probably responsible for T cell chemotaxis. We measured the corresponding receptors of the four screened chemokines on T cells. XCR1 expression in T cells was negligible; therefore, we ruled out XCL1 as a chemoattractant that induces T cell recruitment. XCR1 is a chemokine receptor exclusively expressed on murine and human cross-presenting dendritic cells (DCs) [42]. Moreover, XCL1 specifically attracts CD8^+^ DCs but not CD8^−^ DCs, T cells, B cells, or NK cells [43]. The results of these two studies are consistent with our results. Subsequently, by using blocking experiments, we found that the chemokines CCL1, CCL2, and CCL20 were responsible for attracting T cells. Activated NK cells can produce multiple chemokines, including CCL1, which may recruit other effector cells during immune responses [44]. CCL1 recruits tumor-associated macrophages and Tregs to the tumor niche [45]. Moreover, Cadilha et al. found that CCL1 from activated T cells potentiates a feedback loop for CCR8 + T cell recruitment to the tumor site [46]. These studies support our finding that CCL1 secreted by NK cells recruits T-cells to the tumor site. Previous studies have revealed that CCL2 may cause tumor infiltration by tumor-infiltrating lymphocytes, which have an anticancer effect [47, 48], consistent with our results. As for CCL20, Chew et al. demonstrated that CCL20 could recruit tumor-associated macrophages and resident NK cells to the TME of hepatocellular carcinoma [49]. CCL20 recruits Tregs and Th17 cells to the tumor niche [45]. In addition, Crittenden et al. reported that CCL20 expression in a colorectal tumor model significantly decreased tumorigenesis, which was associated with an increase in CD8 T cells, NK cells, and class II DCs in the tumor [50]. These reports confirmed the role of immune cell chemotaxis of CCL20, which is consistent with our findings. Collectively, our study is the first to systemically and comprehensively clarify the exact chemokine profiles secreted by NK cells responsible for driving T-cell recruitment. Growing evidence suggests that the immune cells, including innate immune cells (macrophages, neutrophils, DCs, innate lymphoid cells, myeloid-derived suppressor cells, and NK cells) and adaptive immune cells (T cells and B cells), influence tumor progression when present in the TME [51]. Huang M, et al. reported that CCL20 secreted by BCa cells can promote macrophage infiltration into the TME [52]. Moreover, Zhang Q, et al. revealed that CXCL1/8 secreted by BCa cells and infiltrating immune cells could recruit tumor-associated neutrophils expressing CXCR1/2 to the TME [53]. Our study suggests that the chemokines secreted by NK and BCa cells during the killing process may recruit other immune cells to the TME except for T cells. The accumulating effects of NK cells killing BCa cells on the reshaping of immune profiles need further exploration.

**Fig. 10:**
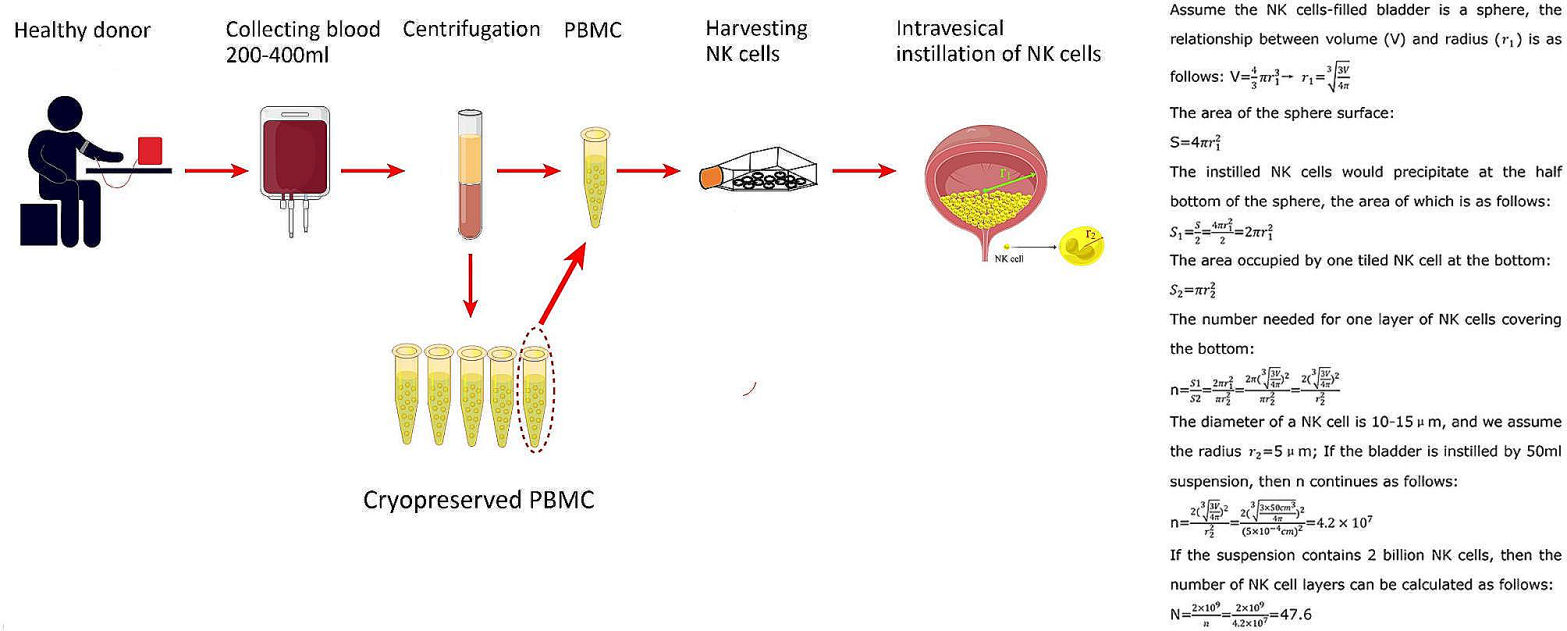
Summary diagram of NK cell production, cryopreservation, and intravesical installation application. Approximately 20–40 billion NK cells can be obtained from one healthy donor’s 200–400 mL of peripheral blood after two weeks of PBMC culture and expansion. The PBMC could be cryopreserved at − 80 °C and applied as an off-the-shelf product for immunotherapy; 47.6 layers of NK cells could be put on the surface of one-half of the bladder wall if 2 billion NK cells suspended in 50 mL of saline are instilled into the bladder. Abbreviations: PBMCs: peripheral blood mononuclear cells

In our study, we obtained large quantities of NK cells using our previous expansion method and ultimately obtained 20–40 billion NK cells from one healthy donor (200–400 mL of peripheral blood) after two weeks of peripheral blood mononuclear cell (PBMC) culture and expansion [21] (Fig. 10). PBMC can be cryopreserved and stored at very low temperatures and can be applied as off-the-shelf products for immunotherapy. After accurate calculation, we found that if we performed intravesical instillation of NK cells at a dose of 2 billion suspended in 50 mL of saline, we could place 47.6 layers of the NK cells on one half of the bladder wall, which was expected to be sufficient to produce potent antitumor and preventive effects against BCa and reduce the recurrence rate after TURBT (Fig. 10). Therefore, the BCa patients after TURBT could receive at least ten times of adjuvant allogeneic NK cell treatment and one time of NK cell therapy included 2 billion NK cells (2 billion/once × 10 times). For example, NK cells can be given once a week for 4 weeks and once a month for 6 months. However, the optimal length and frequency of repeat NK cell instillation need further clinical exploration. Our continued scale-up of the NK cell manufacturing system will enable the banking of these “livable drugs” for on-demand intravesical immunotherapy.

The main limitation of our study is that we evaluated the therapeutic efficacy of human-derived NK cells in a tumor-burden xenograft model using immunocompromised mice lacking human T, B, or NK cells. Thus, the human TME could hardly be simulated in our BCa mouse model. It is well-known that crosstalk between NK cells and DCs via cytokines or direct cell interaction results in activation and cytokine production of both cell types, contributing to the coordination of innate and adaptive immune responses and enhancement of antitumor effects [37, 54, 55]. We believe that the therapeutic effect of NK cell intravesical instillation in immunocompetent patients would be much better than that observed in our immunocompromised mouse model. Future clinical trials and studies using humanized mouse models to reproduce the complex human TME are needed to validate the antitumor clinical efficacy of allogeneic NK cell intravesical instillation immunotherapy.

## Conclusions

We expanded allogeneic NK cells based on our previously established human peripheral NK cell expanding system and validated that NK cells possess high and selective cytotoxicity against BCa cells in vitro and in vivo. We explored the mechanisms underlying the different cytotoxicities of NK cells against BCa and normal urothelial cells. Moreover, we confirmed the chemokine profiles responsible for NK cell-mediated chemotaxis of T cells during the BCa cell-killing process of NK cells. This finding supports the translational application of NK cell instillation with multiple cycles or as maintenance therapy, similar to the strategies used for standard chemotherapy regimens after TURBT from bench to bedside for NMIBC treatment. Our results may bring us to a new and exciting era of NK cell therapy with the potential to change the standards of instillation.

### Electronic supplementary material

Below is the link to the electronic supplementary material.

Supplementary Material 1: RNA analysis of BCa tissue-derived organoid and paracancerous tissue-derived organoid

Supplementary Material 2: The upregulated genes in NK cells cocultured with BCa cells

Supplementary Material 3: The downregulated genes in NK cells cocultured with BCa cells

## Data Availability

The raw data generated in this study are available upon request from the corresponding authors.

## Abbreviations

BCa: Bladder cancer
NMIBC: Non-muscle-invasive bladder cancer
MIBC: Muscle-invasive bladder cancer
TURBT: Transurethral resection of bladder tumor
BCG: Bacillus Calmette-Guérin
NK: Natural killer cells
FBS: Fetal bovine serum
CCL: C-C-motif ligand
CXCL: C-X-C-motif ligand
MHC-I: Major histocompatibility complex class I
MICA/B: MHC-I polypeptide-related sequences A and B
ULBP-2/5/6: UL16-binding protein-2/5/6
KIR: Killer cell Ig-like receptors
PBMCs: Peripheral blood mononuclear cells
AO/PI: Acridine orange/propidium iodide
CCK-8: Cell counting kit-8
E/T: Effector-to-target ratios
OD: Optical density
ELISA: Enzyme-linked immunosorbent assay
IFN: Interferon
TNF: Tumor necrosis factor
IL: Interleukin
PDO: Patient-derived organoids
LDH: Lactate dehydrogenase
HE: Hematoxylin and eosin
SD: Standard deviation
TME: Tumor microenvironment
CAR: Chimeric antigen receptor
TRAIL: TNF-related apoptosis-inducing ligand
CRS: Cytokine release syndrome
DCs: Dendritic cells
