# Supplementary material for: High and selective cytotoxicity of ex vivo expanded allogeneic human natural killer cells from peripheral blood against bladder cancer: implications for natural killer cell instillation after transurethral resection of bladder tumor
Source: J Exp Clin Cancer Res. 2024 Jan 20;43:24. doi: 10.1186/s13046-024-02955-7 (PMC10799482; doi:10.1186/s13046-024-02955-7)
Supplement: Supplementary file 1 — Supplementary Material 1: RNA analysis of BCa tissue-derived organoid and paracancerous tissue-derived organoid [file 13046_2024_2955_MOESM1_ESM.docx]

1. There were 1353 upregulated genes and 1440 downregulated genes when comparing BCa tissue-derived organoid withparacancerous tissue-derived organoid (**Supplementary Fig.1**).


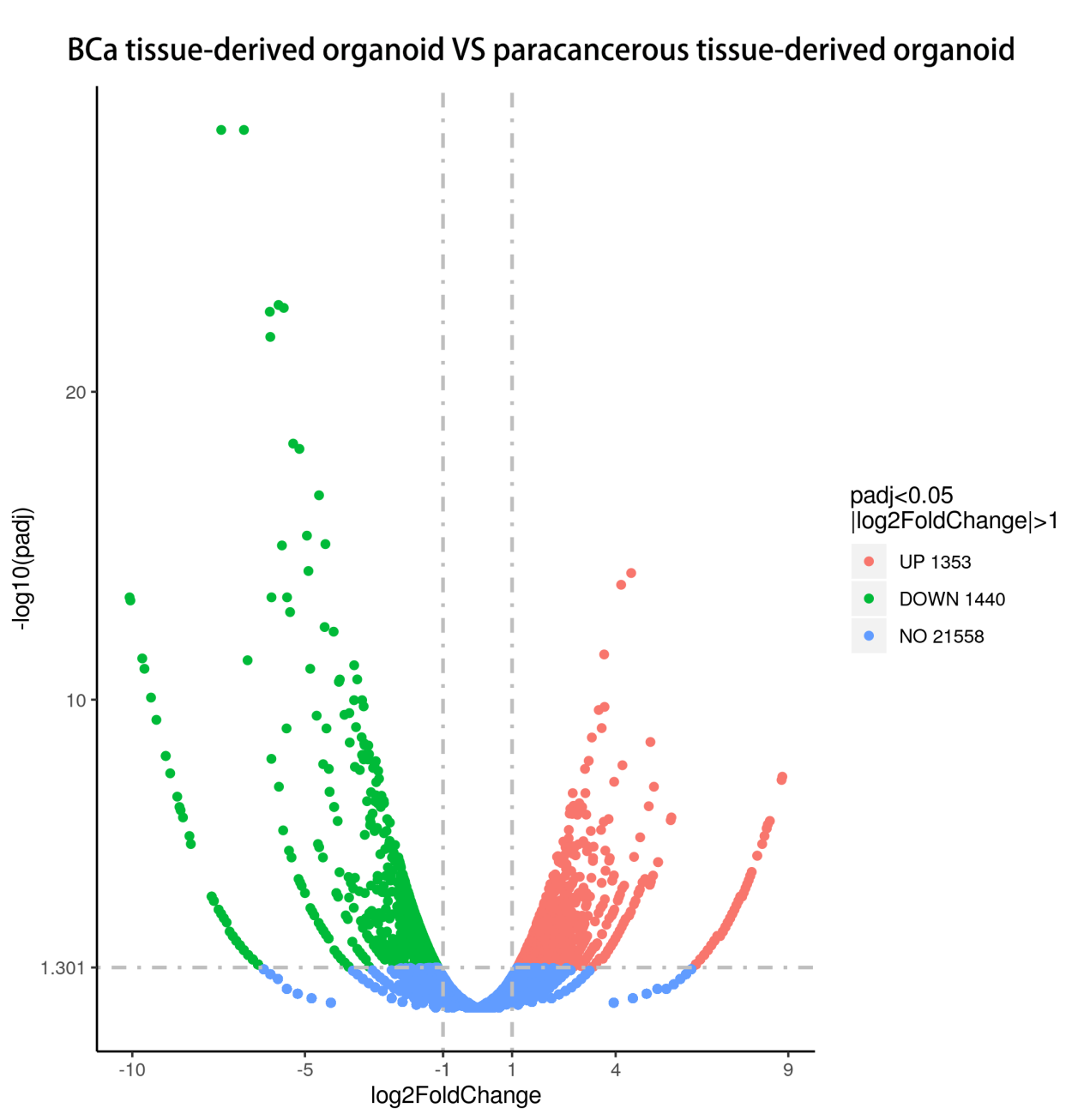


**Supplementary Fig.1** Volcano plots of the differentially expressed genes between BCa tissue-derived organoid and paracancerous tissue-derived organoid, genes differentially expressed with a fold change greater than 1.0 and adjusted p<0.05 are marked in colour. The p-values were calculated using a two-sided unpaired Student’s t-test.

1. The expressions of AC021066.1, AP000346.2, BUB1B, CENPA, CKAP2L, CST1, CST4, FOXM1, LMNB1, MCM2, MKI67, MMP11, RAD51-AS1, TACC3, TOP2A were significantly up-regulated in BCa organoid, when compared the noncancerous counterpart (log_2_Fold Change>2, p < 0.05), which was consistent with the DEG profiles in TCGA Analysis (**Supplementary Table 1**).

**Supplementary Table 1**. TCGA database consistent up-regulated genes in BCa organoid compared the noncancerous counterpart.

| **gene_id** | **BCa tissue-derived organoid** | **Paracancerous tissue-derived organoid** | **log2**  **Fold**  **Change** | **P value** | **padj** | **gene_**  **name** | **gene_**  **chr** | **gene_biotype** | **gene_description** |
| --- | --- | --- | --- | --- | --- | --- | --- | --- | --- |
| ENSG00000257830 | 19.6604201 | 1.39E-17 | 7.31599307 | 3.05E-05 | 0.001244492 | AC021066.1 | 12 | antisense | novel transcript, antisense to KRT86 |
| ENSG00000234353 | 17.8722788 | 1.39E-17 | 7.179394663 | 7.55E-05 | 0.002423447 | AP000346.2 | 22 | transcribed_unprocessed_pseudogene | immunoglobulin lambda-like polypeptide 1 (IGLL1) pseudogene |
| ENSG00000156970 | 16.97820765 | 1.39E-17 | 7.105917838 | 0.000120499 | 0.003396151 | BUB1B | 15 | protein_coding | BUB1 mitotic checkpoint serine/threonine kinase B [Source:HGNC Symbol;Acc:HGNC:1149] |
| ENSG00000115163 | 9.82561877 | 1.39E-17 | 6.325018792 | 0.004174171 | 0.039780651 | CENPA | 2 | protein_coding | centromere protein A [Source:HGNC Symbol;Acc:HGNC:1851] |
| ENSG00000169607 | 18.7663496 | 1.128544151 | 3.92679886 | 0.000376589 | 0.007725629 | CKAP2L | 2 | protein_coding | cytoskeleton associated protein 2 like [Source:HGNC Symbol;Acc:HGNC:26877] |
| ENSG00000170373 | 279.8337362 | 59.28961271 | 2.2366248 | 2.23E-06 | 0.000173806 | CST1 | 20 | protein_coding | cystatin SN [Source:HGNC Symbol;Acc:HGNC:2473] |
| ENSG00000101441 | 12.50784502 | 1.39E-17 | 6.669080841 | 0.00085757 | 0.01364356 | CST4 | 20 | protein_coding | cystatin S [Source:HGNC Symbol;Acc:HGNC:2476] |
| ENSG00000111206 | 49.16466947 | 14.55013921 | 1.74919318 | 0.00374952 | 0.037760367 | FOXM1 | 12 | protein_coding | forkhead box M1 [Source:HGNC Symbol;Acc:HGNC:3818] |
| ENSG00000113368 | 47.37653526 | 11.19468341 | 2.070741133 | 0.001317417 | 0.018771451 | LMNB1 | 5 | protein_coding | lamin B1 [Source:HGNC Symbol;Acc:HGNC:6637] |
| ENSG00000073111 | 75.98667114 | 26.85349542 | 1.4970301 | 0.005055914 | 0.045803126 | MCM2 | 3 | protein_coding | minichromosome maintenance complex component 2 [Source:HGNC Symbol;Acc:HGNC:6944] |
| ENSG00000148773 | 141.2535062 | 16.78711202 | 3.064421904 | 2.29E-08 | 3.95E-06 | MKI67 | 10 | protein_coding | marker of proliferation Ki-67 [Source:HGNC Symbol;Acc:HGNC:7107] |
| ENSG00000099953 | 21.4485603 | 1.39E-17 | 7.440769253 | 1.27E-05 | 0.000658688 | MMP11 | 22 | protein_coding | matrix metallopeptidase 11 [Source:HGNC Symbol;Acc:HGNC:7157] |
| ENSG00000245849 | 221.7194485 | 70.47447685 | 1.652089501 | 0.000393037 | 0.007949201 | RAD51-AS1 | 15 | processed_transcript | RAD51 antisense RNA 1 [Source:HGNC Symbol;Acc:HGNC:48621] |
| ENSG00000013810 | 107.2789926 | 40.27533956 | 1.411097245 | 0.00498578 | 0.045284871 | TACC3 | 4 | protein_coding | transforming acidic coiled-coil containing protein 3 [Source:HGNC Symbol;Acc:HGNC:11524] |
| ENSG00000131747 | 155.5585634 | 31.32744387 | 2.307935157 | 5.80E-06 | 0.000359481 | TOP2A | 17 | protein_coding | DNA topoisomerase II alpha [Source:HGNC Symbol;Acc:HGNC:11989] |

1. The expressions of A2M, ADAMTS9, ADH1B, ANK2, APOD, BVES, C15orf48, C1S, CD69, CHI3L1, CNRIP1, CNTN1, COL14A1, COL6A1, COL6A2, CPED1, CTSV, DCN, DTNA, EDNRA, EDNRB, FABP4, FBLN5,

FENDRR, FGF7, FGL2, FOXF1, GALNT15, GNAL, GPX3, GYPC, HAND2, IL33, IL6, ITIH5, JAM3, KRT14, KRT16, LAMA2, LAMC3, MAD2L1, MASP1, MEG3, NEGR1, PCDH18, PDE1C, PDE1C, PDGFRA, PDLIM3, PEG3, PID1, PRAC1, PRSS8, RBM24, RERG, RGS5, S100A14, SDC2, SFRP1, SFRP2, STEAP4, TCEAL1, TMEM100, TMTC1, TSC22D3, TWIST2, UBE2T, ULBP2, VSTM4, WISP2, ZEB2, ZNF667 were significantly down-regulated in BCa organoid, when compared the noncancerous counterpart (log_2_Fold Change>2, p < 0.05), which was consistent with the DEG profiles in TCGA analysis (**Supplementary Table 2**).

**Supplementary Table 2**. TCGA database consistent down-regulated genes in BCa organoid compared the noncancerous counterpart

| **gene_id** | **BCa tissue-derived organoid** | **Paracancerous tissue-derived organoid** | **log2**  **Fold**  **Change** | **P value** | **padj** | **gene_**  **name** | **gene_**  **chr** | **gene_biotype** | **gene_description** |
| --- | --- | --- | --- | --- | --- | --- | --- | --- | --- |
| ENSG00000175899 | 5.355207028 | 278.5128676 | -5.665749849 | 4.82E-19 | 9.78E-16 | A2M | 12 | protein_coding | alpha-2-macroglobulin [Source:HGNC Symbol;Acc:HGNC:7] |
| ENSG00000163638 | 1.39E-17 | 14.55013921 | -6.883285323 | 0.000517265 | 0.009524599 | ADAMTS9 | 3 | protein_coding | ADAM metallopeptidase with thrombospondin type 1 motif 9 [Source:HGNC Symbol;Acc:HGNC:13202] |
| ENSG00000196616 | 1.39E-17 | 14.55013921 | -6.883285323 | 0.000517265 | 0.009524599 | ADH1B | 4 | protein_coding | alcohol dehydrogenase 1B (class I), beta polypeptide [Source:HGNC Symbol;Acc:HGNC:250] |
| ENSG00000145362 | 1.39E-17 | 12.313168 | -6.644497475 | 0.00143701 | 0.019605563 | ANK2 | 4 | protein_coding | ankyrin 2 [Source:HGNC Symbol;Acc:HGNC:493] |
| ENSG00000189058 | 29.49518184 | 133.1097004 | -2.168801943 | 1.97E-05 | 0.000898631 | APOD | 3 | protein_coding | apolipoprotein D [Source:HGNC Symbol;Acc:HGNC:612] |
| ENSG00000112276 | 8.037461495 | 35.80139193 | -2.13606565 | 0.002205393 | 0.02631236 | BVES | 6 | protein_coding | blood vessel epicardial substance [Source:HGNC Symbol;Acc:HGNC:1152] |
| ENSG00000166920 | 166.287356 | 781.8314833 | -2.23223563 | 5.04E-07 | 5.29E-05 | C15orf48 | 15 | protein_coding | chromosome 15 open reading frame 48 [Source:HGNC Symbol;Acc:HGNC:29898] |
| ENSG00000182326 | 46.4824681 | 126.3987843 | -1.440400751 | 0.002924253 | 0.031676372 | C1S | 12 | protein_coding | complement C1s [Source:HGNC Symbol;Acc:HGNC:1247] |
| ENSG00000110848 | 0.884693594 | 22.37954683 | -4.46532098 | 0.000109596 | 0.003184683 | CD69 | 12 | protein_coding | CD69 molecule [Source:HGNC Symbol;Acc:HGNC:1694] |
| ENSG00000133048 | 1.39E-17 | 104.0290631 | -9.711453422 | 4.50E-15 | 4.57E-12 | CHI3L1 | 1 | protein_coding | chitinase 3 like 1 [Source:HGNC Symbol;Acc:HGNC:1932] |
| ENSG00000119865 | 1.39E-17 | 14.55013921 | -6.883285323 | 0.000517265 | 0.009524599 | CNRIP1 | 2 | protein_coding | cannabinoid receptor interacting protein 1 [Source:HGNC Symbol;Acc:HGNC:24546] |
| ENSG00000018236 | 17.8722788 | 260.617094 | -3.856051752 | 5.08E-13 | 3.09E-10 | CNTN1 | 12 | protein_coding | contactin 1 [Source:HGNC Symbol;Acc:HGNC:2171] |
| ENSG00000187955 | 0.884693594 | 23.49803397 | -4.535330871 | 7.35E-05 | 0.002402777 | COL14A1 | 8 | protein_coding | collagen type XIV alpha 1 chain [Source:HGNC Symbol;Acc:HGNC:2191] |
| ENSG00000142156 | 238.7067021 | 646.4947014 | -1.436850251 | 0.00086864 | 0.013771002 | COL6A1 | 21 | protein_coding | collagen type VI alpha 1 chain [Source:HGNC Symbol;Acc:HGNC:2211] |
| ENSG00000142173 | 62.57567247 | 609.5846699 | -3.281356544 | 5.53E-12 | 2.64E-09 | COL6A2 | 21 | protein_coding | collagen type VI alpha 2 chain [Source:HGNC Symbol;Acc:HGNC:2212] |
| ENSG00000106034 | 0.884693594 | 20.14257265 | -4.314202852 | 0.000248027 | 0.005735705 | CPED1 | 7 | protein_coding | cadherin like and PC-esterase domain containing 1 [Source:HGNC Symbol;Acc:HGNC:26159] |
| ENSG00000136943 | 16.08413613 | 71.59296319 | -2.144577094 | 0.000176356 | 0.004544378 | CTSV | 9 | protein_coding | cathepsin V [Source:HGNC Symbol;Acc:HGNC:2538] |
| ENSG00000011465 | 7.143379861 | 1249.358546 | -7.42381007 | 2.18E-33 | 3.12E-29 | DCN | 12 | protein_coding | decorin [Source:HGNC Symbol;Acc:HGNC:2705] |
| ENSG00000134769 | 4.46111317 | 81.65933978 | -4.153641183 | 1.29E-09 | 3.06E-07 | DTNA | 18 | protein_coding | dystrobrevin alpha [Source:HGNC Symbol;Acc:HGNC:3057] |
| ENSG00000151617 | 2.672897029 | 20.14257265 | -2.851616151 | 0.001084588 | 0.016252802 | EDNRA | 4 | protein_coding | endothelin receptor type A [Source:HGNC Symbol;Acc:HGNC:3179] |
| ENSG00000136160 | 1.39E-17 | 131.9912144 | -10.05457303 | 4.67E-17 | 5.99E-14 | EDNRB | 13 | protein_coding | endothelin receptor type B [Source:HGNC Symbol;Acc:HGNC:3180] |
| ENSG00000170323 | 1485.034578 | 11448.83047 | -2.94651691 | 2.65E-11 | 9.94E-09 | FABP4 | 8 | protein_coding | fatty acid binding protein 4 [Source:HGNC Symbol;Acc:HGNC:3559] |
| ENSG00000140092 | 1.778768091 | 46.98626022 | -4.622326003 | 2.95E-08 | 4.89E-06 | FBLN5 | 14 | protein_coding | fibulin 5 [Source:HGNC Symbol;Acc:HGNC:3602] |
| ENSG00000268388 | 0.884693594 | 26.85349542 | -4.727026919 | 2.30E-05 | 0.001016661 | FENDRR | 16 | lincRNA | FOXF1 adjacent non-coding developmental regulatory RNA [Source:HGNC Symbol;Acc:HGNC:43894] |
| ENSG00000140285 | 0.884693594 | 27.97198256 | -4.785654768 | 1.58E-05 | 0.000764168 | FGF7 | 15 | protein_coding | fibroblast growth factor 7 [Source:HGNC Symbol;Acc:HGNC:3685] |
| ENSG00000127951 | 1.39E-17 | 17.90559876 | -7.180552385 | 0.000120499 | 0.003396151 | FGL2 | 7 | protein_coding | fibrinogen like 2 [Source:HGNC Symbol;Acc:HGNC:3696] |
| ENSG00000103241 | 4.46111317 | 89.48874328 | -4.285552944 | 3.39E-10 | 9.82E-08 | FOXF1 | 16 | protein_coding | forkhead box F1 [Source:HGNC Symbol;Acc:HGNC:3809] |
| ENSG00000131386 | 4.46111317 | 29.09046969 | -2.668212584 | 0.000466301 | 0.008983299 | GALNT15 | 3 | protein_coding | polypeptide N-acetylgalactosaminyltransferase 15 [Source:HGNC Symbol;Acc:HGNC:21531] |
| ENSG00000141404 | 17.8722788 | 155.4794199 | -3.111271428 | 6.08E-09 | 1.19E-06 | GNAL | 18 | protein_coding | G protein subunit alpha L [Source:HGNC Symbol;Acc:HGNC:4388] |
| ENSG00000211445 | 37.54179401 | 378.0581069 | -3.327365184 | 1.59E-11 | 6.73E-09 | GPX3 | 5 | protein_coding | glutathione peroxidase 3 [Source:HGNC Symbol;Acc:HGNC:4555] |
| ENSG00000136732 | 1.39E-17 | 25.73500827 | -7.701084366 | 3.61E-06 | 0.000248026 | GYPC | 2 | protein_coding | glycophorin C (Gerbich blood group) [Source:HGNC Symbol;Acc:HGNC:4704] |
| ENSG00000164107 | 1.39E-17 | 10.07619988 | -6.358191868 | 0.004174171 | 0.039780651 | HAND2 | 4 | protein_coding | heart and neural crest derivatives expressed 2 [Source:HGNC Symbol;Acc:HGNC:4808] |
| ENSG00000137033 | 51.84687055 | 340.0295888 | -2.710115505 | 1.20E-08 | 2.18E-06 | IL33 | 9 | protein_coding | interleukin 33 [Source:HGNC Symbol;Acc:HGNC:16028] |
| ENSG00000136244 | 1.39E-17 | 13.43165334 | -6.768826145 | 0.00085757 | 0.01364356 | IL6 | 7 | protein_coding | interleukin 6 [Source:HGNC Symbol;Acc:HGNC:6018] |
| ENSG00000123243 | 2.672897029 | 19.02408565 | -2.769674305 | 0.001597933 | 0.021216606 | ITIH5 | 10 | protein_coding | inter-alpha-trypsin inhibitor heavy chain family member 5 [Source:HGNC Symbol;Acc:HGNC:21449] |
| ENSG00000166086 | 12.50784502 | 44.74928676 | -1.827420154 | 0.002909732 | 0.031575255 | JAM3 | 11 | protein_coding | junctional adhesion molecule 3 [Source:HGNC Symbol;Acc:HGNC:15532] |
| ENSG00000186847 | 2.672897029 | 33.56441795 | -3.585038621 | 1.06E-05 | 0.000575917 | KRT14 | 17 | protein_coding | keratin 14 [Source:HGNC Symbol;Acc:HGNC:6416] |
| ENSG00000186832 | 86.71546812 | 2093.815319 | -4.591566345 | 8.49E-21 | 2.30E-17 | KRT16 | 17 | protein_coding | keratin 16 [Source:HGNC Symbol;Acc:HGNC:6423] |
| ENSG00000196569 | 16.97820765 | 222.5885746 | -3.702084484 | 5.06E-12 | 2.46E-09 | LAMA2 | 6 | protein_coding | laminin subunit alpha 2 [Source:HGNC Symbol;Acc:HGNC:6482] |
| ENSG00000050555 | 5.355207028 | 41.3938264 | -2.918881929 | 4.11E-05 | 0.00154803 | LAMC3 | 9 | protein_coding | laminin subunit gamma 3 [Source:HGNC Symbol;Acc:HGNC:6494] |
| ENSG00000164109 | 42.01213167 | 171.1382231 | -2.022681183 | 2.80E-05 | 0.001181156 | MAD2L1 | 4 | protein_coding | mitotic arrest deficient 2 like 1 [Source:HGNC Symbol;Acc:HGNC:6763] |
| ENSG00000127241 | 5.355207028 | 52.57869346 | -3.263098502 | 2.52E-06 | 0.000190854 | MASP1 | 3 | protein_coding | mannan binding lectin serine peptidase 1 [Source:HGNC Symbol;Acc:HGNC:6901] |
| ENSG00000214548 | 91.18579982 | 231.5364616 | -1.342965488 | 0.003156945 | 0.033628507 | MEG3 | 14 | lincRNA | maternally expressed 3 [Source:HGNC Symbol;Acc:HGNC:14575] |
| ENSG00000172260 | 0.884693594 | 16.78711202 | -4.05293675 | 0.001369576 | 0.019277765 | NEGR1 | 1 | protein_coding | neuronal growth regulator 1 [Source:HGNC Symbol;Acc:HGNC:17302] |
| ENSG00000189184 | 1.778768091 | 88.37025708 | -5.532006195 | 1.59E-12 | 8.59E-10 | PCDH18 | 4 | protein_coding | protocadherin 18 [Source:HGNC Symbol;Acc:HGNC:14268] |
| ENSG00000154678 | 1.39E-17 | 11.19468341 | -6.508435285 | 0.002434933 | 0.027349193 | PDE1C | 7 | protein_coding | phosphodiesterase 1C [Source:HGNC Symbol;Acc:HGNC:8776] |
| ENSG00000134853 | 4.46111317 | 210.2852298 | -5.517064269 | 3.51E-17 | 4.75E-14 | PDGFRA | 4 | protein_coding | platelet derived growth factor receptor alpha [Source:HGNC Symbol;Acc:HGNC:8803] |
| ENSG00000154553 | 0.884693594 | 24.61652112 | -4.602100037 | 4.96E-05 | 0.001763087 | PDLIM3 | 4 | protein_coding | PDZ and LIM domain 3 [Source:HGNC Symbol;Acc:HGNC:20767] |
| ENSG00000198300 | 0.884693594 | 35.80139193 | -5.140402113 | 9.27E-07 | 8.60E-05 | PEG3 | 19 | protein_coding | paternally expressed 3 [Source:HGNC Symbol;Acc:HGNC:8826] |
| ENSG00000153823 | 1.39E-17 | 14.55013921 | -6.883285323 | 0.000517265 | 0.009524599 | PID1 | 2 | protein_coding | phosphotyrosine interaction domain containing 1 [Source:HGNC Symbol;Acc:HGNC:26084] |
| ENSG00000159182 | 38.43586166 | 111.8584658 | -1.537632651 | 0.002007467 | 0.024882974 | PRAC1 | 17 | protein_coding | PRAC1 small nuclear protein [Source:HGNC Symbol;Acc:HGNC:30591] |
| ENSG00000052344 | 978.9932796 | 2367.844338 | -1.274076895 | 0.002409836 | 0.027349193 | PRSS8 | 16 | protein_coding | serine protease 8 [Source:HGNC Symbol;Acc:HGNC:9491] |
| ENSG00000112183 | 1.778768091 | 23.49803397 | -3.626125873 | 0.000348534 | 0.007322821 | RBM24 | 6 | protein_coding | RNA binding motif protein 24 [Source:HGNC Symbol;Acc:HGNC:21539] |
| ENSG00000134533 | 8.037461495 | 42.51231321 | -2.383205087 | 0.000411667 | 0.00823706 | RERG | 12 | protein_coding | RAS like estrogen regulated growth inhibitor [Source:HGNC Symbol;Acc:HGNC:15980] |
| ENSG00000143248 | 58.99940553 | 280.7498393 | -2.247847634 | 1.52E-06 | 0.000127028 | RGS5 | 1 | protein_coding | regulator of G protein signaling 5 [Source:HGNC Symbol;Acc:HGNC:10001] |
| ENSG00000189334 | 697.3625203 | 2308.564591 | -1.726811466 | 5.05E-05 | 0.001790327 | S100A14 | 1 | protein_coding | S100 calcium binding protein A14 [Source:HGNC Symbol;Acc:HGNC:18901] |
| ENSG00000169439 | 13.40191869 | 74.94842215 | -2.47138035 | 2.06E-05 | 0.000935954 | SDC2 | 8 | protein_coding | syndecan 2 [Source:HGNC Symbol;Acc:HGNC:10659] |
| ENSG00000104332 | 0.884693594 | 102.910577 | -6.660706181 | 5.39E-15 | 5.25E-12 | SFRP1 | 8 | protein_coding | secreted frizzled related protein 1 [Source:HGNC Symbol;Acc:HGNC:10776] |
| ENSG00000145423 | 2.672897029 | 27.97198256 | -3.323068066 | 8.17E-05 | 0.002578368 | SFRP2 | 4 | protein_coding | secreted frizzled related protein 2 [Source:HGNC Symbol;Acc:HGNC:10777] |
| ENSG00000127954 | 176.1220824 | 796.3717986 | -2.175983354 | 8.39E-07 | 7.98E-05 | STEAP4 | 7 | protein_coding | STEAP4 metalloreductase [Source:HGNC Symbol;Acc:HGNC:21923] |
| ENSG00000172465 | 177.9102145 | 454.1151426 | -1.351192832 | 0.001926501 | 0.024169106 | TCEAL1 | X | protein_coding | transcription elongation factor A like 1 [Source:HGNC Symbol;Acc:HGNC:11616] |
| ENSG00000166292 | 1.39E-17 | 25.73500827 | -7.701084366 | 3.61E-06 | 0.000248026 | TMEM100 | 17 | protein_coding | transmembrane protein 100 [Source:HGNC Symbol;Acc:HGNC:25607] |
| ENSG00000133687 | 1.778768091 | 42.51231321 | -4.478336552 | 9.80E-08 | 1.33E-05 | TMTC1 | 12 | protein_coding | transmembrane and tetratricopeptide repeat containing 1 [Source:HGNC Symbol;Acc:HGNC:24099] |
| ENSG00000157514 | 877.9638327 | 2486.403832 | -1.501674844 | 0.000377965 | 0.007740807 | TSC22D3 | X | protein_coding | TSC22 domain family member 3 [Source:HGNC Symbol;Acc:HGNC:3051] |
| ENSG00000233608 | 0.884693594 | 20.14257265 | -4.314202852 | 0.000248027 | 0.005735705 | TWIST2 | 2 | protein_coding | twist family bHLH transcription factor 2 [Source:HGNC Symbol;Acc:HGNC:20670] |
| ENSG00000077152 | 50.95280356 | 190.1524837 | -1.897024663 | 6.29E-05 | 0.002135949 | UBE2T | 1 | protein_coding | ubiquitin conjugating enzyme E2 T [Source:HGNC Symbol;Acc:HGNC:25009] |
| ENSG00000131015 | 222.6135145 | 625.2434712 | -1.48927822 | 0.000578848 | 0.010288701 | ULBP2 | 6 | protein_coding | UL16 binding protein 2 [Source:HGNC Symbol;Acc:HGNC:14894] |
| ENSG00000165633 | 2.672897029 | 21.26105972 | -2.929152959 | 0.000739506 | 0.01242095 | VSTM4 | 10 | protein_coding | V-set and transmembrane domain containing 4 [Source:HGNC Symbol;Acc:HGNC:26470] |
| ENSG00000064205 | 24.13076895 | 135.3466724 | -2.480988278 | 2.19E-06 | 0.000171836 | WISP2 | 20 | protein_coding | WNT1 inducible signaling pathway protein 2 [Source:HGNC Symbol;Acc:HGNC:12770] |
| ENSG00000169554 | 5.355207028 | 45.8677735 | -3.066559285 | 1.44E-05 | 0.00072466 | ZEB2 | 2 | protein_coding | zinc finger E-box binding homeobox 2 [Source:HGNC Symbol;Acc:HGNC:14881] |
| ENSG00000198046 | 1.778768091 | 16.78711202 | -3.143731753 | 0.005247305 | 0.046845236 | ZNF667 | 19 | protein_coding | zinc finger protein 667 [Source:HGNC Symbol;Acc:HGNC:28854] |
